# Diagnostic accuracy of point-of-care natriuretic peptide testing for chronic heart failure in ambulatory care: systematic review and meta-analysis

**DOI:** 10.1136/bmj.k1450

**Published:** 2018-05-21

**Authors:** Kathryn S Taylor, Jan Y Verbakel, Benjamin G Feakins, Christopher P Price, Rafael Perera, Clare Bankhead, Annette Plüddemann

**Affiliations:** Nuffield Department of Primary Care Health Sciences, University of Oxford, Oxford OX2 6GG, UK

## Abstract

**Objective:**

To assess the diagnostic accuracy of point-of-care natriuretic peptide tests in patients with chronic heart failure, with a focus on the ambulatory care setting.

**Design:**

Systematic review and meta-analysis.

**Data sources:**

Ovid Medline, Cochrane Central Register of Controlled Trials, Cochrane Database of Systematic Reviews, Database of Abstracts of Reviews of Effects, Embase, Health Technology Assessment Database, Science Citation Index, and Conference Proceedings Citation Index until 31 March 2017.

**Study selection:**

Eligible studies evaluated point-of-care natriuretic peptide testing (B-type natriuretic peptide (BNP) or N terminal fragment pro B-type natriuretic peptide (NTproBNP)) against any relevant reference standard, including echocardiography, clinical examination, or combinations of these, in humans. Studies were excluded if reported data were insufficient to construct 2×2 tables. No language restrictions were applied.

**Results:**

42 publications of 39 individual studies met the inclusion criteria and 40 publications of 37 studies were included in the analysis. Of the 37 studies, 30 evaluated BNP point-of-care testing and seven evaluated NTproBNP testing. 15 studies were done in ambulatory care settings in populations with a low prevalence of chronic heart failure. Five studies were done in primary care. At thresholds >100 pg/mL, the sensitivity of BNP, measured with the point-of-care index device Triage, was generally high and was 0.95 (95% confidence interval 0.90 to 0.98) at 100 pg/mL. At thresholds <100 pg/mL, sensitivity ranged from 0.46 to 0.97 and specificity from 0.31 to 0.98. Primary care studies that used NTproBNP testing reported a sensitivity of 0.99 (0.57 to 1.00) and specificity of 0.60 (0.44 to 0.74) at 135 pg/mL. No statistically significant difference in diagnostic accuracy was found between point-of-care BNP and NTproBNP tests.

**Conclusions:**

Given the lack of studies in primary care, the paucity of NTproBNP data, and potential methodological limitations in these studies, large scale trials in primary care are needed to assess the role of point-of-care natriuretic peptide testing and clarify appropriate thresholds to improve care of patients with suspected or chronic heart failure.

## Introduction

An estimated 800 000 people in the UK currently have heart failure, with more than 250 000 new cases every year. Incidence increases with age and is highest in adults aged more than 75 years. The aging population and improved survival of people with ischaemic heart disease are likely to lead to a continuing increase in the prevalence of heart failure. Overall, a general practitioner with a patient population of 2000 will care for about 40-50 patients with heart failure and see two or three new cases each year.^1^

Since heart failure may be reversible with appropriate treatment in the early stages of disease, early diagnosis is important. Considering the low prevalence of heart failure, however, general practitioners are unlikely to have enough experience to identify more subtle signs. Although heart failure is often diagnosed by general practitioners, on the basis of clinical signs, symptoms, and the results of 12 lead electrocardiography, the diagnosis is only confirmed by echocardiography in about one third of cases.^2^ Cardiologists generally perform better than general practitioners in using electrocardiography to rule out chronic heart failure (CHF); however, electrocardiography is not a reliable test to diagnose CHF because of its non-specific nature.^3^ ^4^ Therefore, recent guidelines from the National Institute for Health and Clinical Excellence, and the European Society for Cardiology (ESC) on the initial diagnosis of CHF and referral for echocardiography recommend the use of B-type natriuretic peptide (BNP) tests in combination with clinical assessment.^5^ ^6^

BNP is a protein produced by heart muscle cells as a prohormone (proBNP) and released into the cardiovascular system in response to ventricular dilation and pressure overload.^7^ The prohormone is split by a protease and secreted as the physiologically active C terminal fragment (BNP) and the inactive N terminal fragment (NTproBNP), which has a longer half life than BNP.

No clear consensus exists about the appropriate BNP threshold for diagnosing CHF. The 2016 ESC guidelines recommend an upper limit of normal of 35 pg/mL for BNP and 125 pg/mL for NTproBNP in the non-acute setting to exclude heart failure. In the acute setting, higher values should be used (BNP <100 pg/mL, NTproBNP <300 pg/mL).^6^ In contrast, NICE suggests a BNP threshold of 100 pg/mL and an NTproBNP threshold of 400 pg/mL for referral of patients with suspected heart failure.^5^

Traditionally, hospital laboratories have carried out BNP testing, typically taking up to a day to return results. Many laboratories offer slightly quicker turnaround times but point-of-care BNP tests give results within minutes. The use of point-of-care devices in primary care and other ambulatory care settings allows BNP results to be available when acute management decisions are needed. As well as reducing turnaround time, point-of-care testing by general practitioners could lead to a quicker investigation of dyspnoea, more timely referral, earlier initial treatment, and less uncertainty and anxiety for patients. The development of point-of-care natriuretic peptide testing services in community settings is part of a general effort to move care from hospital settings to the community and make more point-of-care tests available for a range of conditions.^8^ Several point-of-care devices that test for BNP or NTproBNP are available.

Systematic reviews have been done on the role and accuracy of BNP and NTproBNP testing in the diagnosis of CHF^3^ ^9^ ^10^ ^11^ ^12^ ^13^ ^14^; however, none has focused specifically on the diagnostic accuracy of point-of-care testing. We reviewed the diagnostic accuracy of point-of-care natriuretic peptide tests in patients with suspected or confirmed CHF, with a focus on ambulatory care settings.

## Methods

### Search strategy

Our search strategy (see appendix 1 for protocol) was based on a combination of subject headings and terms for heart failure, the two natriuretic peptides, point-of-care testing (including “point-of-care”, “rapid”, “same time”, “immediate”, “bed-side”), and the known point-of-care index devices (“Triage”, “Cardiac Reader”, “Abbott iSTAT”, “RAMP”, “Cobas h232”, “Alere Heart Check”). We searched several electronic databases from inception until 31 March 2017: Ovid Medline (see appendix 2), Cochrane Central Register of Controlled Trials, Cochrane Database of Systematic Reviews, Database of Abstracts of Reviews of Effects, Embase, Health Technology Assessment Database, Science Citation Index, and Conference Proceedings Citation Index. To maximise the sensitivity of the search strategy we did not use filters for diagnostic studies. For unpublished trials, we searched ClinicalTrials.gov and the trials registers on the World Health Organization International Clinical Trials Registry Platform. We also used the “related articles” feature in PubMed and searched reference lists of included studies to identify more publications.

### Selection of studies

Two reviewers (KST, AP, CB; in pairs) screened titles and abstracts of the search results independently, and disagreements were resolved by referral to a third reviewer. Studies of point-of-care BNP and NTproBNP tests were included that reported diagnostic accuracy as an outcome and compared the index test result with that of any relevant reference standard, including echocardiography, clinical examination, or combinations of these. We excluded comparisons of laboratory based reference tests as they compared methods rather than diagnostic accuracy. Both prospective and retrospective case-control and cohort trial studies were included. Although the focus of the review was the ambulatory care setting, we included studies with participants with suspected or confirmed CHF in all settings because what is considered ambulatory care can vary between countries and we wanted to include all studies with relevant populations. We defined ambulatory care as primary, outpatient, or emergency care—all settings where ambulatory patients with heart failure would present—with the aim of investigating the potential use of point-of-care BNP tests in primary care. We imposed no restrictions on study population numbers or language—studies in languages other than English were translated—and study quality was not an exclusion criterion. Letters, narrative reviews, and other non-primary sources were excluded. Studies were also excluded if they did not provide sufficient data to construct a 2×2 table, were based on animals or non-human samples, or presented duplicate data. If two publications had overlapping populations, they were counted as a single study, and we only included both in our study if each provided diagnostic accuracy data at different thresholds. If both only provided data at the same thresholds, we excluded the publication with the smaller population and recorded this as a duplicate study.

### Data extraction and management

Two reviewers (KT, CB, JV, AP; in pairs) extracted data independently using a predefined data extraction sheet (see appendix 3), cross checked the data, and resolved disagreements by discussion or referral to a third reviewer. If more than one publication with overlapping populations reported diagnostic accuracy data at the same threshold, we only extracted data from the publication with the larger population.

### Assessment of methodological quality

Two reviewers (KT, CB, AP, in pairs) independently assessed the quality of included studies and this was cross checked by a third reviewer (JV), using QUADAS-2.^15^

### Data analysis and synthesis

We extracted binary diagnostic accuracy data from all studies and constructed 2×2 tables at all thresholds. To obtain an overview of test accuracy, we produced a receiver operating characteristic plot of sensitivity and specificity estimates for all thresholds for each natriuretic peptide test. In these plots, we split the data by index test and study design. Study design was defined as either case-control or cross sectional/cohort. Case-control studies were those described as such and also studies with selected healthy and unhealthy populations. As the results of case-control studies are susceptible to bias, we excluded these studies from further analysis that split the data by population (prevalence of CHF) and setting. A study population was categorised as having a high (>50%) or low (≤50%) prevalence of CHF. Study setting was grouped as ambulatory (primary, outpatient, or emergency care), mixed outpatient and inpatient, or inpatient only.

For each natriuretic peptide, we produced paired forest plots with corresponding 95% confidence intervals. We grouped data into categories based on the thresholds recommended by NICE^5^: <100 pg/mL and ≥100 pg/mL for BNP, and <400 pg/mL and ≥400 pg/mL for NTproBNP.

We then analysed ambulatory care settings only and populations with a low prevalence of heart failure to ensure generalisability to primary care patients. To establish adherence to the thresholds recommended in ESC and NICE guidelines, we summarised the thresholds used in the included studies and pooled data at the recommended thresholds, where possible. We also compared the diagnostic accuracy of BNP and NTproBNP tests.

Summary receiver operating characteristic curves and forest plots were plotted using R version 3.4.2 and RevMan version 5.3.

To generate pooled estimates of sensitivity and specificity, we applied bivariate meta-analysis methods.^16^ We used hierarchical summary receiver operating characteristic meta-analysis methods to produce summary receiver operating characteristic curves with corresponding 95% confidence regions and prediction regions for cross sectional/cohort studies collectively for BNP and NTproBNP. Where studies reported data at multiple thresholds, as these were overlapping data, we selected the lowest threshold only for each study because this was consistent with our focus on ambulatory rather than inpatient care. The xtmelogit command in STATA SE version 14.2 was used for these analyses, and parameters were entered directly into RevMan to produce Cochrane standardised output.^17^

We analysed data from studies with laboratory based reference tests separately from data from studies with reference tests based on clinical assessment. We also analysed data based on populations with suspected CHF separately from data based on populations with confirmed CHF.

To assess heterogeneity between studies, we visually inspected the summary receiver operating characteristic curves and forest plots. We also adjusted for possible sources of heterogeneity by adding covariates to the bivariate model and testing with likelihood ratio tests, and we did a subgroup analysis.^17^ ^18^ Both approaches focused on concerns about patient selection and used QUADAS-2 assessments. A study was classified as having concerns if either the risk of bias or the applicability concerns was assessed as high, or if both were assessed as unclear. For the analysis adjusting for possible sources of heterogeneity we created a binary variable (with or without concerns about patient selection), and for the subgroup analysis we excluded studies with concerns about patient selection from the comparison of the diagnostic accuracy between BNP and NTproBNP.

To assess the robustness of our conclusions in our comparison of the diagnostic accuracy of BNP and NTproBNP testing, we evaluated the effect of removing outliers for all studies in ambulatory care settings and using a different data pooling method. This was an extension of the bivariate method of Dukic and Gatsonis,^19^ which permits the inclusion of multiple threshold values for each study. This was done with R, and using simulation we calculated pointwise confidence intervals for sensitivity and specificity at given thresholds. The selected thresholds were those given in the ESC and NICE guidelines.

To test for publication bias, we applied the Deeks’ test,^20^ where data allowed, although in cases of high heterogeneity, this would result in low power.^20^ ^21^ ^22^ In reporting our results, the term “accuracy” is often used to refer to diagnostic accuracy.

### Patient involvement

Members of a patient and public involvement group were part of the Stakeholder group and Steering Committee of the National Institute for Health Research (NIHR) programme grant that funded this study. The patient and public involvement group includes people with heart failure and other lay people. Updates and details about the study were presented to the steering committee while the study was ongoing, and the public members provided feedback. Two members of this group commented on our completed manuscript. No patients nor patient representatives were involved in setting the outcome measures, nor were they directly involved in developing plans for the design or implementation of the study. A one day dissemination event is planned to report the results of all the studies funded by the NIHR programme grant, including this study. Members of the patient and public involvement group will be invited to this event.

## Results

**Fig 1:**
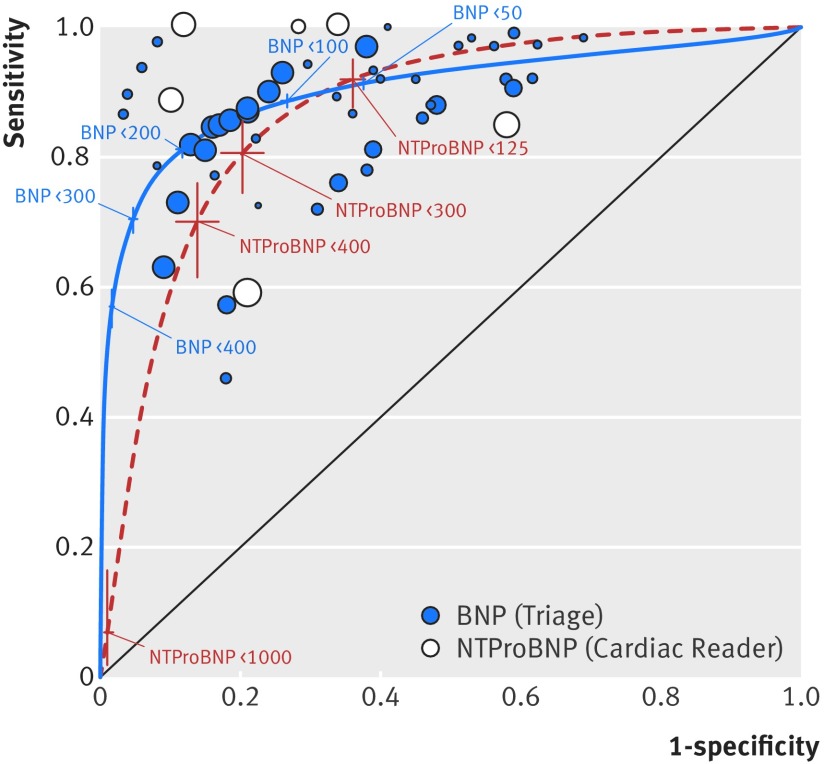
Flow diagram of study selection. Two studies were reported by more than one publication

Figure 1 summarises the search results and the inclusion and exclusion of studies. We identified 2604 references through database and registry searches, and an additional 17 publications were identified by checking reference lists of retrieved reviews and using the “related articles” function in PubMed. After removing duplicates, we screened 878 records by title and abstract. Of these and the references identified through reference list searches, the full text of 116 records was reviewed, resulting in 42 publications of 39 individual studies that met our inclusion criteria. The references of all 42 publications are given in appendix 4.

Five of the studies were in a primary care setting (two assessed BNP and three assessed NTproBNP).^23^ ^24^ ^25^ ^26^ ^27^ Two studies gave data for confirmed CHF cases.^28^ ^29^ Seven studies had a case-control design, and 32 were cross sectional/cohort studies, 15 of which had populations presenting mainly with dyspnoea, six had populations with several signs and symptoms of CHF, five had populations of echocardiography referrals, two had populations with cardiovascular risk factors, two had populations with stable CHF referred for cardiac rehabilitation, one had a population with acute coronary embolism, and one had a non-cardiac population receiving mechanical ventilation (see supplementary table S1). Most of the included studies were in secondary care settings, the majority in the emergency department (table 1). The prevalence of CHF in the studies in primary care ranged from 19% to 44%. Of the 14 studies in emergency care settings, 11 reported a prevalence of CHF at or below 50%. The reference test in most of the studies was clinical assessment with retrospective review by one doctor or more (usually cardiologists). Some studies used echocardiography alone as the reference test (table 2).

**Table 1:** Included studies, study design and setting, and population characteristics

Studies	Design	No of participants	Prevalence of heart failure (%)*	Setting	Age (years)†	Men (%)
**Studies included in analysis**						
Ajuluchukwu 2009	Case-control	72 (42 inpatients+30 controls)	58 - high	Inpatients and controls were staff and escorts, Nigeria	>14	Not stated
Alibay 2005	Cross sectional/cohort	160	38 - low	Emergency department, France	80.1 (13.5)	48
Blondé-Cynober 2011	Cross sectional/cohort	64	41 - low	Inpatients, France	84.3 (7.4)	31
Breathing not properly study:						
Maisel 2002	Cross sectional/cohort	1586	47 - low	Emergency department, International	64 (17)	56
Maisel 2003	Cross sectional/cohort	1586	47 - low	Emergency department, International	64 (17)	56
Pahle 2009‡	Cross sectional/cohort	1583 (740 elevated blood pressure+843 normal blood pressure)	47 - low	Emergency department, International	Elevated: 67 (54-78); normal: 64 (49-76)	Elevated: 51.8; normal: 60
Chenevier-Gobeaux 2010	Cross sectional/cohort	378	30 - low	Emergency department, France	78 (12)	50
Dao 2001	Cross sectional/cohort	250	39 - low	Emergency and urgent care departments, USA	63 (0.86)	94
De Vecchis 2016	Cross sectional/cohort	111§	44 - low	Outpatients, Italy	58 (47-65)	65
Dokanish 2004	Cross sectional/cohort	122	57 - high	Inpatients, USA	56 (13)	51
Fischer 2001	Cross sectional/cohort but similar to case-control	145 (95 cardiac+50 healthy)	29 - low	Unclear, Germany	Cardiac: 61.9 (20-60)¶; healthy range: 19-86	Cardiac: 67.4; healthy: 60
Fuat 2006	Cross sectional/cohort	297	38 - low	One-stop diagnostic clinics in 2 hospitals and general practices, England	73.8 (34-94)¶	37
Gorissen 2007	Cross sectional/cohort	80	50 - low	Emergency department, Netherlands	74 (10)	55
Gruson 2009	Cross sectional/cohort	97	20 - low	Emergency department, Belgium	71 (30-95)	57
Jungbauer 2012	Case-control	222 (151 confirmed+71 healthy)	16, 24, 22, or 38†† - low	Outpatients and controls were healthy hospital employees, Germany	Confirmed: 62.9 (12.1); healthy: 39.7 (15.1)	Confirmed: 71.5; healthy: 40.8
Knudsen 2004	Cross sectional/cohort	155	48 - low	Emergency department, Norway	Men: 74 (66-79); women: 78 (71-84)	44.5
San Diego veterans’ study:						
Krishnaswamy 2001	Cross sectional/cohort	400	63 - high	Outpatients and inpatients, USA	65.7 (12.2)	96
Lubien 2002	Cross sectional/cohort	294	40 - low	Outpatients and inpatients, USA	64.5 (5.5)	90
Lainchbury 2003	Cross sectional/cohort	205	34 - low	Emergency department, New Zealand	70 (14)	49
Logeart 2002**	Cross sectional/cohort	163	71 - high	Inpatients, France	67.4 (14.8)	67
Maisel 2001	Cross sectional/cohort	200	48 - low	Inpatients and outpatients, USA	65.3 (0.9)	95
Mak 2004	Cross sectional/cohort	100	16 - low	Inpatients and outpatients, USA	64 (13)	97
Monfort 2015	Cross sectional/cohort	163§	69 - high (class II-IV)	Cardiac rehabilitation, France	Median 58	81
Prontera 2005	Cross sectional/cohort but similar to case-control	284 (214 confirmed+91 healthy)	57 - high (of 213)	Unclear, Italy	Confirmed 62 (13); healthy 43.2 (13.4)	Confirmed 77; healthy 44
Prosen 2011	Cross sectional/cohort	218	59 - high	Prehospital emergency, Slovenia	63.3 (16.1)	71
Ro 2011	Cross sectional/cohort	250	43 - low	Emergency department, USA	70.7 (13.8)	57.8
Shao 2005	Cross sectional/cohort	103	61 - high	Unclear, China	Not stated	Not stated
Storti 2004	Cross sectional/cohort but similar to case-control	296 (202 cardiac+94 healthy)	59 - high (of 227)	Cardiac inpatients, Italy	Cardiac: 59.3 (20.5); healthy: 43.5 (14)	Cardiac: 70.3; healthy: 39.4
Su 2015	Cross sectional/cohort	268	56 - high (of 203)	Emergency department, China	All 74.1 (7.9)	All 56.3
Tang 2005	Case-control	348 (241 confirmed+107 healthy)	69 - high	Secondary care, USA	Confirmed: male 69.4, female 69.1; Normal: male 44.0, female 44.9	Not stated
Taylor 2017	Cross sectional/cohort	304	34 - low	Primary care, England	73.9 (8.8)	40.8
Tomonaga 2011	Cluster randomised controlled trial	369 (218 in POCT group)	44 - low (of 70 from POCT group)	Primary care, Switzerland	POCT group 65 (16)	POCT group 57.9
Verdu 2012	Cross sectional/cohort	220	24 - low	Primary care, Spain	73.2 (19.2)	34.5
Villacorta 2002	Cross sectional/cohort	70	51 - high	Emergency department, Brazil	72.4 (15.9)	47
Watson 2016	Cross sectional/cohort	1368 (966 diabetes, 402 no diabetes)	19 - low	Primary care, Ireland	Diabetes: 65.7 (58.6-71.6); no diabetes: 67.9 (59.5-74.4)	Diabetes: 64.9; no diabetes: 47.0
Weekes 2016	Cross sectional/cohort	116	22 - low	Emergency department, USA	59 (26)	51
Wei 2005	Cross sectional/cohort	135	45 - low	Outpatients, China	67.8 (11.9)	63
Wieczorek 2002	Case-control	1050 (409 cardiac+641 controls)	39 - low	Inpatients and outpatients, USA	Not stated	Not stated
Zapata 2014	Cross sectional/cohort	86	58 - high	Inpatients, Spain	63.8 (12.7)	66.3
Zhao 2008	Cross sectional/cohort	195	69 - high	Inpatients, China	72.1 (8.3)	51.8
**Eligible studies not included in analysis**						
Morrison 2002	Cross sectional/cohort	321	42 - low	Emergency department, USA	Not stated	Not stated
Vanderheyden 2006	Cross sectional/cohort	72	56 - high	Inpatients, Belgium	65 (12)	71

POCT=point-of-care testing.

* As defined by reference standard, which, if based on clinical assessment, could use a single test or multiple tests.

† Mean (SD), or median (interquartile range) unless stated otherwise.

‡ Reported baseline characteristics in groups based on blood pressure and hypertension history—numbers refer to patients with blood pressure status recorded.

§ All with confirmed heart failure.

¶ Mean (range).

** Arrivals at emergency department, but 90% were later admitted to intensive care.

†† Evaluated diagnostic accuracy using four different definitions of heart failure: New York Heart Association classes III and IV, left ventricular ejection fraction <40%, fluid retention, and American College of Cardiology/American Heart Association stages C and D, respectively.

**Table 2:** Included studies, point-of-care tests and thresholds, and reference tests

Studies	Point-of-care tests*	Thresholds (pg/mL)	Reference tests
**Studies included in analysis**			
Ajuluchukwu 2009	Cardiac Reader (NTproBNP)	95, 100, 105, 110, 113, 115, 200, 122, 124, 125, 126, 127, 130, 135, 140, 145	Clinical evaluation and echocardiography. Evaluation of cases and controls by study assistant, senior registrar, or investigator
Alibay 2005	Triage (BNP)	50, 100, 150, 200	Retrospective review by two senior cardiologists
Blondé-Cynober 2011	Triage (BNP)	18, 100, 129, 400, 635	Retrospective review by cardiologist and geriatrician
Breathing not properly study:			
Maisel 2002	Triage (BNP)	50, 80, 100, 125, 150	Retrospective review by two cardiologists
Maisel 2003	Triage (BNP)	Additional 200, 300, 400	Retrospective review
Pahle 2009	Triage (BNP)	Additional 120, 140, 160, 180	Retrospective review
Chenevier-Gobeaux 2010	Triage (BNP)	100	Retrospective review by two senior emergency physicians
Dao 2001	Triage (BNP)	80, 100, 115, 120, 150	Retrospective review by two cardiologists
De Vecchis 2016	Alere (BNP)	412	New York Heart Association classification
Dokanish 2004	Triage (BNP)	250	Retrospective review by cardiologist
Fischer 2001	Triage (BNP)	130	Echocardiography
Fuat 2006	Triage (BNP).	40, 100	Echocardiography
Gorissen 2007	Triage (BNP)	78, 225, 260, 309	Retrospective review by cardiologist and pulmonologist
Gruson 2009	Biosite SOB panel (BNP)	100	Retrospective review
Jungbauer 2012	Cardiac Reader (NTproBNP); Triage (BNP)	410; 117	Based on clinical signs, physical examination, and echocardiography
Knudsen 2004	Triage (BNP)	50, 100, 150, 200	Retrospective review by two cardiologists
San Diego veterans’ study:			
Krishnaswamy 2001	Triage (BNP)	49, 62, 75, 110, 160, 345	Retrospective review of echocardiography, admission treatment for heart failure, and visits to the emergency department for heart failure
Lubien 2002	Triage (BNP)	Additional 17.5, 62, 92, 130	Echocardiography
Lainchbury 2003	Triage (BNP)	69, 104, 208, 277, 346	Retrospective review by two cardiologists, with third cardiologist as adjudicator
Logeart 2002	Triage (BNP)†	80, 100, 150, 200, 250, 300, 400	Retrospective review by two cardiologists and pneumologist
Maisel 2001	Triage (BNP)	38.5, 46, 55, 65, 75	Echocardiography
Mak 2004	Triage (BNP)	90, 173, 279, 402	Echocardiography
Monfort 2015	Alere (BNP)	159	New York Heart Association classification
Prontera 2005	Triage (BNP)	5.1, 29	Retrospective review
Prosen 2011	Cardiac Reader (NTproBNP)	1000	Retrospective review by cardiologists or intensive care physicians, or both
Ro 2011	Triage (BNP); Abbott i-STAT (BNP)	100; 100	Based on discharge diagnosis, echocardiography (when available), and assessment of a consulting cardiologist
Shao 2005	Triage (BNP)	100	Echocardiography, and cardiac catheterization
Storti 2004	Triage (BNP)	40.7	Based on clinical (presence of suggestive symptoms), and echocardiographic evidence
Su 2015	RAMP (NTproBNP)	600	Retrospective review
Tang 2005	Triage (BNP)	52, 74, 100	Retrospective review
Taylor 2017	Cardiac Reader (NTproBNP)	125, 400	Retrospective review by expert panel
Tomonaga 2011	Cardiac Reader (NTproBNP)	125	Retrospective review
Verdu 2012	Cardiac Reader (NTproBNP)	125, 280, 400	Retrospective review by one cardiologist
Villacorta 2002	Triage (BNP)	200	Retrospective review by one cardiologist
Watson 2016	Triage (BNP)	10, 15, 25, 30, 50	Echocardiography
Weekes 2016	Abbott i-STAT (BNP)	90	Echocardiography
Wei 2005	Triage (BNP)	40	Retrospective review by two cardiologists
Wieczorek 2002	Triage (BNP)	100	Retrospective review by one physician
Zapata 2014	Triage (BNP)	125, 100	Echocardiography
Zhao 2008	Triage (BNP)	50, 80, 100, 130, 150	Cardiac catheterisation
**Eligible studies not included in analysis**			
Morrison 2002	Triage (BNP)	94, 105, 135, 195, 240	Retrospective review by two cardiologists
Vanderheyden 2006	Triage (BNP)	29.3, 50, 100, 139	Cardiac catheterisation

NTproBNP=N terminal fragment pro B-type natriuretic peptide; BNP=B-type natriuretic peptide; SOB=shortness of breath.

* Point-of-care devices: Cardiac Reader/Cobas h 232 (Roche Diagnostics); Triage (Biosite Diagnostics); RAMP (Response Biomedical Corporation), Abbott i-STAT (Abbott Point of Care); Alere^TM^ Heart Check (Alere).

† Two point-of-care tests (Triage, to measure BNP, and Hewlett Packard Sonos 1500, to provide Doppler echocardiography) were compared with the same reference test to indirectly compare BNP with Doppler echocardiography. Only point-of-care test is listed in table.

Various thresholds were used for the index tests, ranging from 5.1 to 412 pg/mL for BNP and 117 to 1000 pg/mL for NTproBNP (table 2). Thirty three studies reported data for BNP and seven for NTproBNP. Of the studies that gave diagnostic accuracy statistics at unique threshold values, the most common threshold for BNP was 100 pg/mL (15 of 33 studies)—the BNP threshold recommended by NICE and ESC (acute setting).^5^ ^6^ The most common threshold for NTproBNP was 125 pg/mL (4 of 7 studies)—the threshold recommended by ESC (non-acute setting). No studies reported BNP data at 35 pg/mL—the BNP threshold recommended by ESC (non-acute setting), and none reported NTproBNP data at 300 pg/mL—the threshold recommended by ESC (acute setting). Two studies reported NTproBNP data at 400 pg/mL—the threshold recommended by NICE.

### Methodological quality of included studies

**Fig 2:**
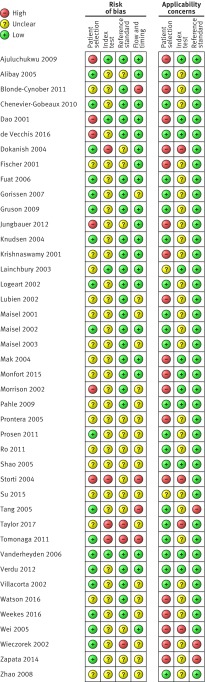
QUADAS-2 summary of risk of bias and applicability concerns showing review authors’ judgments about each domain for each included study. Based on 42 publications (39 studies)

**Fig 3:**
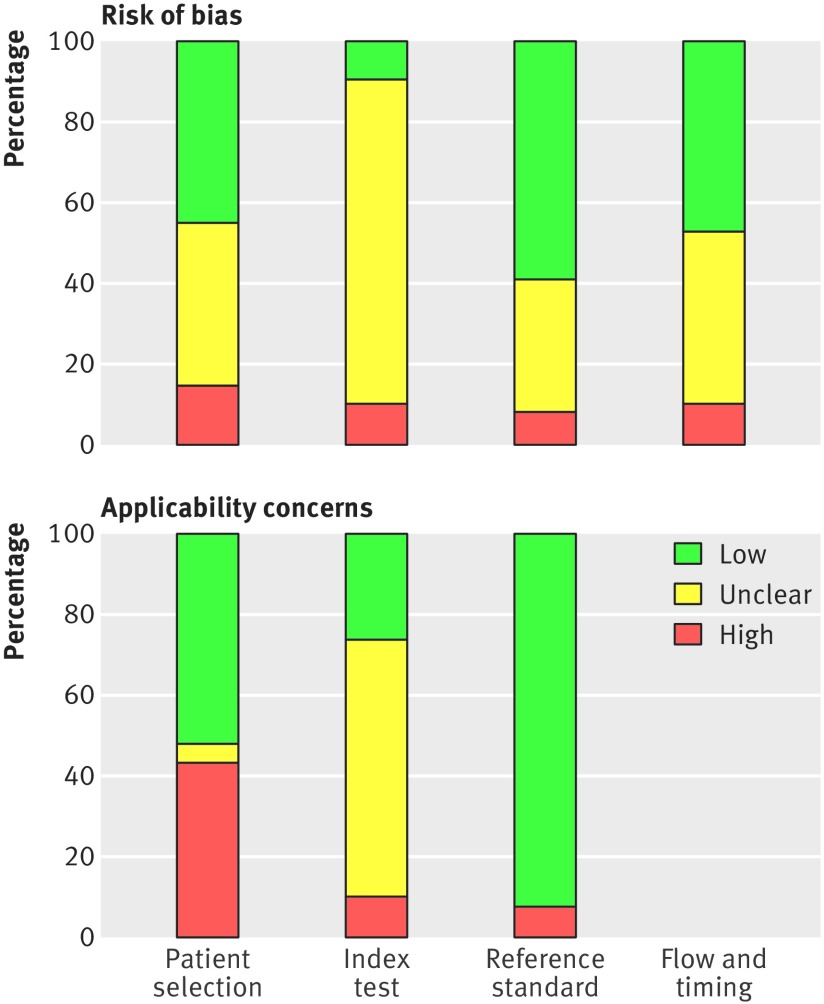
QUADAS-2 risk of bias and applicability concerns graph showing review authors’ judgments about each domain as percentages of included studies. Based on 42 publications (39 studies)

All included studies were assessed using the QUADAS-2 framework. Figures 2 and 3 summarise the overall risk of bias and applicability concerns. For patient selection, the risk of bias overall was generally low as most studies included consecutive series of patients with suspected or confirmed CHF. However, applicability concerns were high with respect to the question of this systematic review, because the patients were often not representative of an ambulatory care population. The risk of bias for the index test was generally unclear because in most studies it was not obvious whether the thresholds used had been prespecified, with some using study derived thresholds, and whether the index test was performed blinded to the results of the reference test. Applicability concerns for the index test were also considered unclear because of blinding as it was not obvious how the point-of-care natriuretic tests would perform if interpreted without knowing the results of the reference tests, which would be the case if they were done in ambulatory and primary care settings as part of a diagnostic investigation. The risk of bias and applicability concerns for the reference standard were both assessed as low because most studies used an appropriate reference standard—clinical examination or echocardiography, or both. Flow and timing was rated as low risk of bias; however, several studies were rated as unclear risk of bias because the time interval between tests was often not explicitly reported.

We found no evidence of publication bias (see supplementary fig S1). The number of studies included in the analysis was, however, small so the power to detect bias was low.

Our analysis of the diagnostic accuracy of the peptides was based on data from 40 publications of 37 individual studies that met our inclusion criteria and provided usable data (fig 1).

### B-type natriuretic peptide

**Fig 4:**
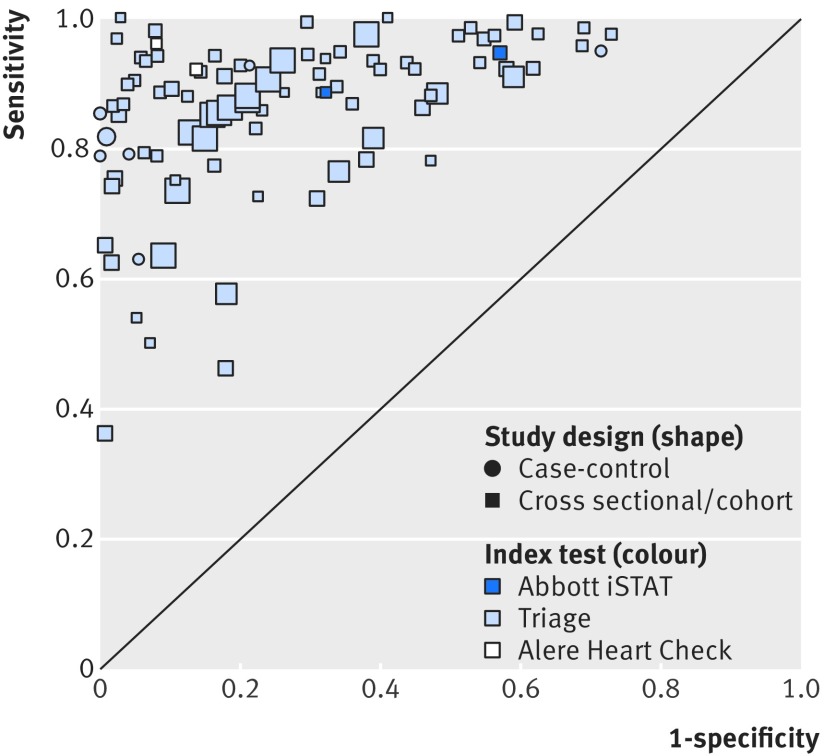
Receiver operating characteristic plot of B-type natriuretic peptide compared with clinical assessment, grouped by study design and index test for all thresholds. Based on data for 29 studies (32 publications). Size of symbol indicates study size

Figure 4 provides an overview of the results from 32 publications reporting data from 29 individual studies on the accuracy of point-of-care BNP tests compared with clinical assessment, grouped by study design and index test manufacturer for all thresholds. Most studies assessed the accuracy of the Triage test (light blue symbols) compared with clinical assessment using a cross sectional/cohort design (squares). Two studies reported the accuracy of the Abbott iSTAT test (dark blue symbols) and two reported the accuracy of the Alere Heart Check test in patients with confirmed CHF (white symbols). The reported sensitivity and specificity varied considerably between studies. The lowest sensitivity came from one study on a primary care population with very low prevalence of CHF (19%)^27^ and two studies on inpatient and outpatient populations that used BNP thresholds >345 pg/mL.^30^ ^31^

**Fig 5:**
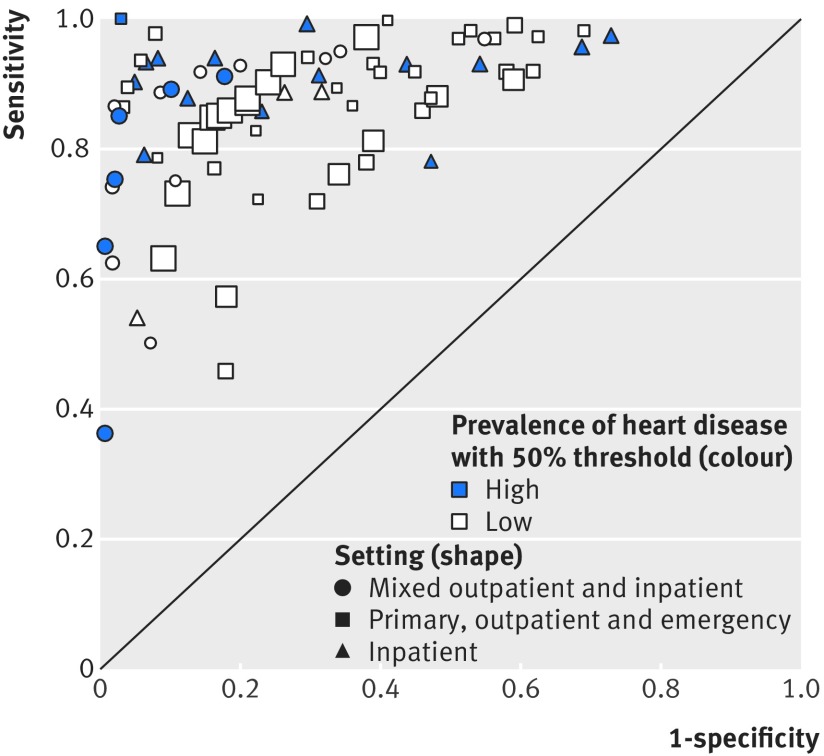
Receiver operating characteristic plot of B-type natriuretic peptide compared with clinical assessment, grouped by setting and prevalence of heart failure, for cross sectional/cohort studies and all thresholds. Based on data for 22 studies (25 publications). All index tests were Triage. Size of symbol indicates study size. Excludes one study (Shao 2005) because setting was not clear

The accuracy of the point-of-care BNP test in populations with a high (dark blue symbols) compared with a low (white symbols) prevalence of CHF in ambulatory care settings (squares) varied considerably between studies and at different thresholds (fig 5).

For the cross sectional/cohort studies in ambulatory care settings that had populations with a low prevalence of CHF, the two primary care studies had a CHF prevalence of 19% and 38%, the outpatient study a prevalence of 45%, and the 12 emergency care studies a prevalence ranging from 20% to 50%.

**Fig 6:**
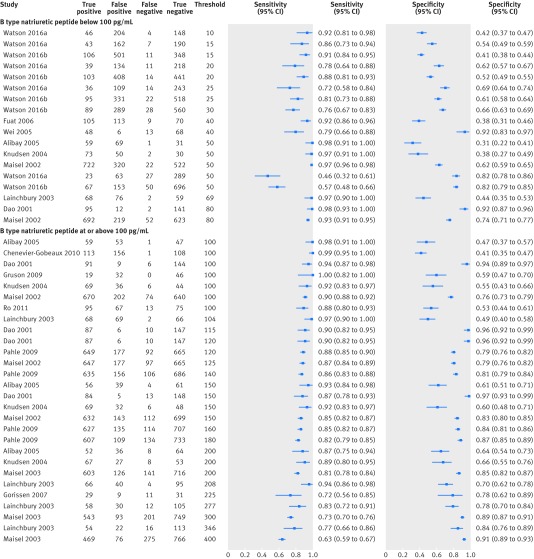
Paired sensitivity and specificity plots at two threshold levels for B-type natriuretic peptide compared with clinical assessment, for cross sectional/cohort studies with populations of low prevalence of chronic heart failure in ambulatory care settings. Based on data for 13 studies (15 publications). All index tests were Triage. Watson 2016a refers to a population with diabetes and Watson 2016b to a population without diabetes

The sensitivity of BNP testing in these studies was slightly less variable than the specificity, and sensitivity decreased as the threshold increased (fig 6). Of note, at thresholds <100 pg/mL, several studies reported low sensitivities and specificities (<0.8) of the test, and variations in sensitivity and specificity did not appear to correlate with increasing threshold. At a threshold of <100 pg/mL, sensitivity ranged from 0.46 (95% confidence interval 0.32 to 0.61) to 0.98 (0.93 to 1.00) and specificity from 0.31 (0.22 to 0.41) to 0.98 (0.95 to 1.00). The two studies^23^ ^27^ in a primary care setting reported sensitivities ranging from 0.46 (0.32 to 0.61) to 0.92 (0.81 to 0.98) and specificities from 0.38 (0.31 to 0.46) to 0.82 (0.79 to 0.85) at a range of thresholds <100 pg/mL (10-50 ng/mL). The study by Watson et al,^27^ which reported the lowest sensitivity, included a population with a low prevalence of CHF (19%) as it included patients with risk factors for cardiovascular disease, not with symptoms of CHF.

When we pooled data at and around the thresholds recommended by the ESC and NICE guidelines for studies in ambulatory care settings and with a low prevalence of CHF (see supplementary table S2), the sensitivity of BNP was highest at 100 pg/mL (recommended by NICE for all settings and by ESC for acute settings^5^ ^6^): pooled sensitivity 0.95 (95% confidence interval 0.90 to 0.98) and pooled specificity 0.64 (0.46 to 0.78). This was based on seven studies in emergency settings with a prevalence of CHF between 20% and 48%.

Only one cross sectional/cohort study^32^ had a high prevalence of CHF in an ambulatory care setting and reported a sensitivity of 1.00 (0.90 to 1.00) and specificity of 0.97 (0.95 to 1.00) at 100 pg/mL threshold for the Triage test.

In the few cross sectional/cohort studies with a low prevalence of CHF in mixed inpatient and outpatient settings (see supplementary fig S2), variations in sensitivity and specificity correlated with increasing threshold.

### N terminal fragment pro B-type natriuretic peptide

**Fig 7:**
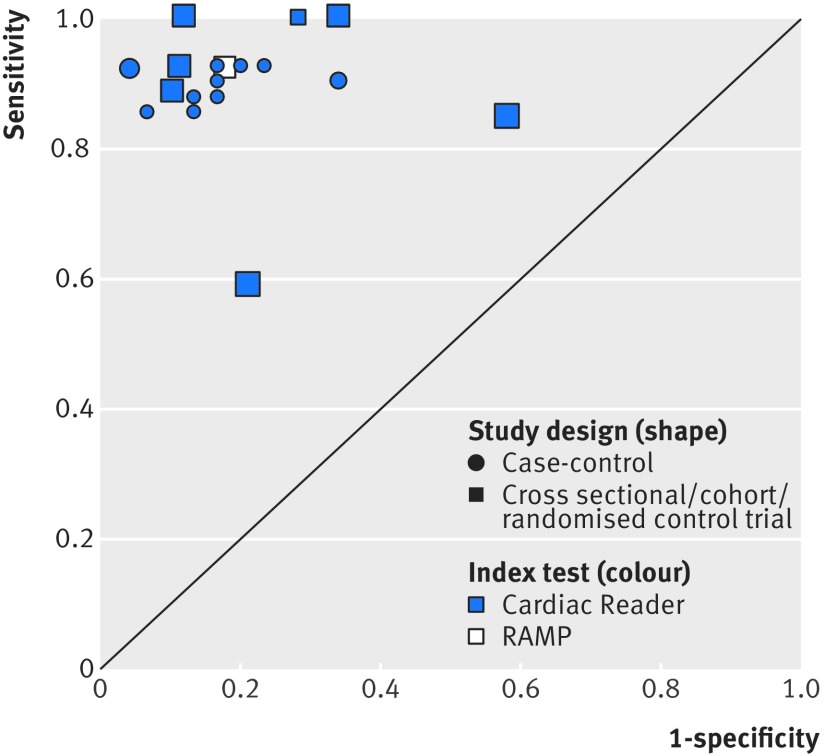
Receiver operating characteristic plot of N terminal fragment pro B-type natriuretic peptide compared with clinical assessment, grouped by study design and index test for all thresholds. Based on data for seven studies. Size of symbol indicates study size

Most NTproBNP studies reported results for the Cardiac Reader NTproBNP test compared with clinical assessment. Figure 7 shows the results from seven individual studies reporting the accuracy of point-of-care NTproBNP testing compared with clinical assessment, grouped by study design and index test manufacturer for all reported thresholds. Two index tests were evaluated: the Cardiac Reader (Roche; six studies, blue symbols) and RAMP (Response Biomedical; one study, white symbol). Three of these studies were done in a primary care setting.^24^ ^25^ ^26^ Overall, sensitivity and specificity were less variable than those reported for BNP, with the exception of one of the primary care studies,^24^ where the reported sensitivity and specificity were lower than those reported in other studies. For this study, a risk of bias was identified for the reference test and patients were preselected using a clinical decision rule.

**Fig 8:**
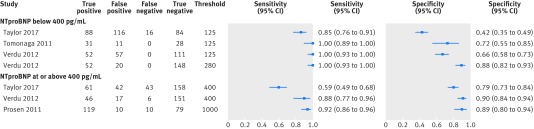
Paired sensitivity and specificity plots at two thresholds levels for N terminal fragment pro B-type natriuretic peptide compared with clinical assessment, for cross sectional/cohort/randomised controlled trial studies. Based on data for four studies. All index tests were Cardiac Reader

In studies in ambulatory care settings with a cross sectional/cohort design at 125 pg/mL, the threshold recommended by ESC for non-acute settings, the paired sensitivity and specificity plots for the Cardiac Reader test show high sensitivity (see supplementary table S2, pooled sensitivity based on three primary care studies,^24^ ^25^ ^26^ 0.99 (95% confidence interval 0.57 to 1.00)) and moderate specificity (pooled specificity 0.60 (0.44 to 0.74)), and increased specificity at higher thresholds (fig 8, supplementary table S2). At 400 pg/mL (the threshold recommended by NICE), in the two studies done in a primary care setting, sensitivity ranged from 0.59 (0.49 to 0.68) to 0.88 (0.77 to 0.96) and specificity from 0.79 (0.73 to 0.84) to 0.90 (0.84 to 0.94). Sensitivity and specificity were consistently high in the case-control studies (see supplementary fig S3).

### Comparison of diagnostic accuracy of the two peptides

**Fig 9:**
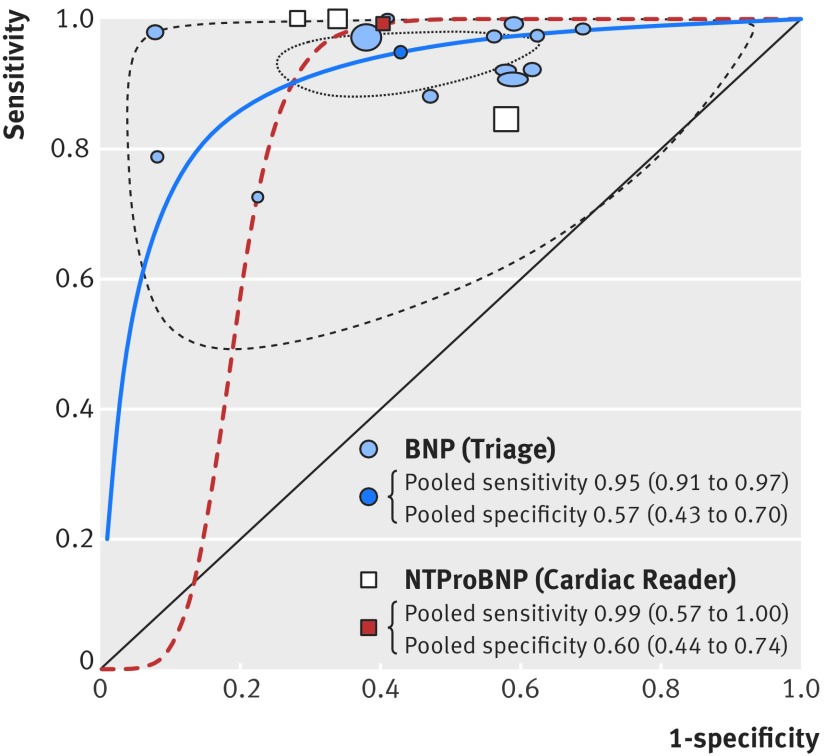
Summary receiver operating characteristic plots for B-type natriuretic peptide (BNP) and N terminal fragment pro B-type natriuretic peptide (NTproBNP) compared with clinical assessment, for cross sectional/cohort/randomised controlled trial studies of populations with low prevalence of heart failure in primary, outpatient, and emergency settings and the lowest threshold for each study. Based on data for 15 studies, 3 NTproBNP and 12 BNPs. Size of symbol indicates study size. Studies were insufficient to draw meaningful prediction and confidence regions for NTproBNP

The summary receiver operating characteristic plots assessing BNP and NTproBNP tests, each compared with clinical assessment and at the lowest threshold for each study, showed that NTproBNP was slightly more accurate than BNP (fig 9), but the difference was not statistically significant (Triage (BNP) pooled sensitivity 0.95 (95% confidence interval 0.92 to 0.97), pooled specificity 0.57 (0.43 to 0.70); Cardiac Reader (NTproBNP) pooled sensitivity 0.97 (0.57 to 1.00), pooled specificity 0.69 (0.44 to 0.74)). The confidence and prediction regions were wide for NTproBNP because of the lack of data (not shown).

Removing the outlier^24^ did not alter this result (Cardiac Reader (NTproBNP) pooled sensitivity 0.99 (0.75 to 1.00), pooled specificity 0.77 (0.62 to 0.87)). When emergency care studies with a high prevalence of CHF were also included, there was also no significant difference (see supplementary fig S4). Sensitivities remained high, with sensitivity slightly higher for NTproBNP, and confidence intervals overlapped.

**Fig 10:**
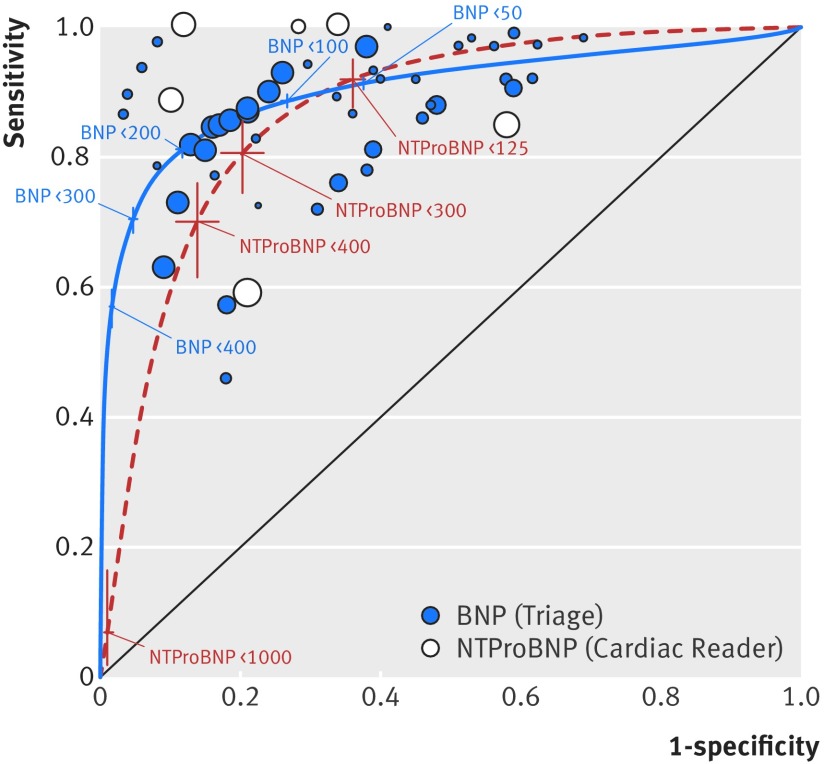
Summary receiver operating characteristic plots for B-type natriuretic peptide (BNP) and N terminal fragment pro B-type natriuretic peptide (NTproBNP) compared with clinical assessment, for cross sectional/cohort studies/randomised controlled trial studies of populations with low prevalence of heart failure in primary, outpatient, and emergency settings and at all thresholds. Based on data for 18 publications (15 studies), 3 NTproBNP and 12 BNP. Size of symbol indicates study size

At all thresholds, the diagnostic accuracy of BNP and NTproBNP was comparable (fig 10). The summary receiver operating characteristic plots were similar and the confidence intervals generally overlapped. BNP points clustered around the point estimate of sensitivity 0.85 and specificity 0.80, whereas NTproBNP points were more scattered because of lack of data.

Because of concerns about patient selection, we tested for heterogeneity between studies using BNP data in the cross sectional/cohort studies with a low prevalence of CHF and in ambulatory care settings. NTproBNP data were insufficient for testing. Four of the 15 studies either had a high risk of bias or had applicability concerns about patient selection, or both were uncertain. We did not find evidence of heterogeneity between studies (likelihood ratio test result, P=0.4) for patient selection. The power to detect heterogeneity was low because of the small number of studies included in the analysis.

After excluding the four studies for which we had concerns about patient selection, we again compared the diagnostic accuracy of the two peptides at the lowest threshold for each study. The pooled sensitivity and specificity were not significantly different, with sensitivity only slightly higher and NTproBNP still slightly more accurate (Triage BNP: pooled sensitivity 0.96 (95% confidence interval 0.90 to 0.98), pooled specificity 0.50 (0.39 to 0.60)).

## Discussion

In populations with a low prevalence of chronic heart failure (CHF) in ambulatory care settings, the diagnostic accuracy of peptide testing varied and data in primary care settings were limited. In emergency care populations with a low prevalence of CHF, the diagnostic accuracy of B-type natriuretic peptide (BNP) measured with the point-of-care device Triage at thresholds at and above 100 pg/mL was similar to that in an acute setting, with sensitivity generally high. Sensitivity was highest at 100 pg/mL, the threshold recommended in the NICE and ESC guidelines (acute setting) (0.95, 95% confidence interval 0.90 to 0.98), and specificity varied. However, in the two primary care studies that reported BNP at and below thresholds of 50 pg/mL, sensitivity and specificity varied widely.

Only five of the 15 studies in ambulatory care settings were done in primary care, and the prevalence of CHF varied (19% to 44%).

Fewer studies assessed the accuracy of N terminal fragment pro B-type natriuretic peptide (NTproBNP). Based on three studies in primary care that used the Cardiac Reader, the sensitivity of NTproBNP was high at 125 pg/mL, the ESC recommended threshold for non-acute settings (0.99, 95% confidence interval 0.57 to 1.00).

No statistically significant difference was found in diagnostic accuracy between point-of-care BNP and NTproBNP testing.

### Strengths and limitations of this study

Our study provides a comprehensive review of the current evidence on the diagnostic accuracy of point-of-care BNP and NTproBNP tests in the diagnosis of CHF in ambulatory care at all thresholds. However, a limitation of our analysis was the few data in primary care settings and the few data for NTproBNP. The lack of data also limited the power of our statistical tests. The risk of bias assessment highlighted that in several studies it was unclear whether assessors interpreting the results of the point-of-care index test (BNP or NTproBNP) were blinded to the outcome of the reference test (eg, clinical assessment, echocardiography). The lack of blinding may lead to an overestimation of the test accuracy.^33^ Therefore, if these tests are used in general practice without a reference test, their performance is still not known. Furthermore, the heterogeneity in assessment of the reference standard across studies might affect generalisability of these findings to settings with different diagnostic protocols for patients with CHF, which would result in a different specificity of the test used. To ensure generalisability to primary care patients, we focused on studies in ambulatory care settings—primary, outpatient, and emergency—where ambulatory patients with heart failure would present and populations would have a low prevalence of heart failure. Although some patients arriving at the emergency department will not be ambulatory, many of the included studies supported the inclusion of low risk emergency settings as an ambulatory setting by excluding high risk cases (acute coronary syndrome, acute myocardial infarction, stroke, unstable angina, pulmonary embolism, pneumothorax, pleural effusion, and trauma). Our sensitivity analysis showed that adding emergency care studies with populations with a higher prevalence of CHF did not alter our conclusion that testing for NTproBNP was slightly more accurate than testing for BNP to exclude CHF, which added support for including emergency settings in our analysis. Given our data limitations and the heterogeneity between studies, we cannot draw firm conclusions about appropriate thresholds; instead we summarise the data where possible and present some tentative conclusions.

### Comparison with previous findings

Previous research on the diagnostic accuracy of BNP and NTproBNP include reviews published in 2007, 2008, and 2015. The 2007 review by Clerico et al analysed studies with paired measurement of NTproBNP and BNP in the emergency department and found no difference.^9^ Similar results were reported in the 2008 meta-analysis by Worcester et al.^14^ The diagnostic accuracy of BNP and NTproBNP testing was also found to be similar in the recent 2015 review by Roberts at al.^12^ All three reviews focused on studies carried out in the hospital setting, and they included laboratory tests. Our review of point-of-care tests included 11 studies from the 2015 review; of these, nine were in an ambulatory care setting. We included and analysed data from 12 more ambulatory care studies. We used a meta-analysis method that allowed the use of all available data at different thresholds, thereby providing a comprehensive analysis.^19^ The 2015 review, which assessed the diagnostic accuracy of natriuretic peptides for heart failure in the acute setting, concluded that the use of BNP and NTproBNP testing at the thresholds of the 2012 ESC guideline had excellent ability to exclude a diagnosis of acute heart failure because reported sensitivities were sufficiently high (approaching 1).^12^ The sensitivities of point-of-care tests in the ambulatory care setting assessed in our review were more variable than in the acute setting, particularly in the primary care setting.

### Implications for clinical practice

As the sensitivity in the primary care setting is variable, it is unclear whether point-of-care tests could be used to exclude CHF in primary care. This variability could be because patients with CHF in primary care often present with non-specific symptoms, such as breathlessness. Furthermore, the generally low specificity of these tests in primary care may limit their use as a test to help confirm CHF in primary care, although the NTproBNP test may perform slightly better than the BNP test as a test to rule out CHF because of its higher sensitivity and less variability reported in most studies.

As with any test, the results need to be interpreted in the context of general clinical assessment. If the clinical presentation clearly indicates CHF or a different diagnosis, clinical judgment should overrule a single point-of-care test result.

The limited data from studies in a primary care setting suggest that the diagnostic accuracy of point-of-care BNP and NTproBNP testing at lower thresholds is insufficient. It should also be noted that the thresholds used in many of the studies are likely to be too high to be applicable to primary care. However, some studies reported a high sensitivity, particularly for NTproBNP to exclude CHF at the ESC threshold of 135 pg/mL in non-acute care, which suggests that this threshold might be appropriate for point-of-care testing. This finding is only tentative, given the limitations of our data. As with peptide testing in hospitals, point-of-care testing in ambulatory care would need to follow established clinical guidelines by confirming positive NTproBNP test results with cardiac imaging and ensuring an appropriate safety net through follow-up appointments.^5^ ^6^

### Implications for future research

Given the lack of studies in a primary care setting and potential methodological limitations in the studies that have been done, large scale trials in primary care are needed to assess the role of point-of-care natriuretic peptide testing in improving the care of patients with heart failure and its effect on patient outcomes, such as morbidity and mortality.

### Conclusions

In ambulatory care settings in populations with a low prevalence of CHF, the sensitivity of BNP point-of-care tests at 100 pg/mL, the threshold recommended by NICE and ESC for acute care, is high but this threshold may not be appropriate for the primary care setting specifically. At lower thresholds, including the ESC recommended threshold for non-acute care of 35 pg/mL, results in primary care settings vary. Testing for NTproBNP might be slightly better than testing for BNP to exclude CHF, and the ESC threshold for non-acute care may be appropriate for NTproBNP point-of-care testing; however, prospective trials would need to confirm this. Point-of-care testing, supported by confirmatory testing such as ultrasound imaging, might improve the management of patients with CHF in ambulatory care.

**What is already known on this topic**

Owing to improved survival rates, the prevalence of heart failure will continue to increase; if detected early, heart failure may be reversible with appropriate treatment
Point-of-care testing for natriuretic peptides in primary care and other ambulatory care settings could enable the identification and rapid referral of patients with heart failure, and align with the increasing drive to move care from hospital settings to the community
National Institute for Health and Clinical Excellence and European Society for Cardiology (ESC) guidelines recommend testing for natriuretic peptides to aid the diagnosis of heart failure, and have recommended thresholds to exclude heart failure, but the diagnostic accuracy at these thresholds in ambulatory care is unknown

**What this study adds**

B-type natriuretic peptide has variable ability to exclude chronic heart failure in patients in ambulatory care at low thresholds, but the ESC threshold for non-acute care for N terminal pro B-type natriuretic peptide might be an appropriate cut-off for point-of-care testing in this setting
As with peptide testing in acute care, given the variability of the specificity of natriuretic peptides, clinical guidelines must be followed and all positive test results confirmed by cardiac imaging and appropriately followed up

## Web extra

Extra material supplied by authors

**Supplementary information:** Additional tables, figure, and references

## Notes

Contributors: AP, CB, and RP conceived and designed the study. AP and CB did the initial search, and KST, AP, and CB did the updated search. KST, AP, and CB selected studies for inclusion. KST, AP, CB, and JYV extracted data. KST did the data analysis with advice from RP and JYV. JYV and CPP gave clinical advice in interpreting the results. BGF produced the R plots and prepared the figures for journal submission. KST, AP, CB, and JYV wrote the first draft of the manuscript, and all authors contributed critically to subsequent revisions and approved the final manuscript. KST had full access to all data in the study and takes responsibility for the integrity and accuracy of the data analysis. KST and AP are guarantors. The corresponding author attests that all listed authors meet authorship criteria and that no others meeting the criteria have been omitted.

Ethical approval: Not required.

Data sharing: No additional data available.

Transparency: The lead author and manuscript’s guarantor (KST) affirms that the manuscript is an honest, accurate, and transparent account of the study being reported; that no important aspects of the study have been omitted; and that any discrepancies from the study as planned have been explained.
