# Supplementary material for: Diagnostic accuracy of point-of-care natriuretic peptide testing for chronic heart failure in ambulatory care: systematic review and meta-analysis
Source: BMJ. 2018 May 21;361:k1450. doi: 10.1136/bmj.k1450 (PMC5960954; doi:10.1136/bmj.k1450)
Supplement: Supplementary file 1 — Supplementary information: Additional tables, figure, and references [file tayk042797.ww1.pdf]

SUPPLEMENTARY MATERIAL

Table S1. Inclusion and exclusion criteria

Reference	Inclusion criteria	Exclusion criteria
Ajuluchukwu 2009	Study: Inpatients with new or decompensated HF, satisfying Framingham criteria for HF, with clinical evaluation and echocardiography within 24 hours of admission, aged over 14 years, with absence of other significant pulmonary and liver disease, evidence of cardiac disease demonstrated as cardiac enlargement, serum creatinine <160 µmol/L<br>Controls (staff or patient escorts): Aged over 14 years, non-smoker, absence of cardiopulmonary, renal, and liver disease, non-pregnant females, normal results for serum creatinine and a normal echocardiogram.	Study patients: Current or recent history of smoking, significant history of chronic pulmonary, liver, or current renal impairment, defined as serum creatinine level >160 µmol/L.<br>Controls: current and recent smoking history, clinical evidence or past history of significant cardiopulmonary, liver, renal and liver disease, abnormal renal function, defined by creatinine >120 µmol/L, and abnormal echocardiogram.
Alibay 2005	Admissions for acute dyspnea.	None stated.
Blonde-Cynober 2011	Hospitalized in Emile-Roux geriatric hospital and presenting suspected HF	None stated.
Chenevier-Gobeaux 2010	Aged over 60 years. Dyspneic patients attending the emergency department between the hours of 1700 and 0830.	None stated.
Dao 2001	Presenting to the urgent-care area of the San	Patients whose dyspnea was clearly not secondary

	Diego Veteran's Health Care System with symptoms of dyspnea with shortness of breath as a prominent complaint. Associated symptoms could be edema, weight gain, cough or wheezing.	to CHF (trauma or cardiac tamponade) were excluded. Patients with acute coronary syndromes were excluded unless their predominant presentation was CHF.
De Vecchis 2016	With stable CHF referred to two cardiac rehabilitation centers	None stated.
Dokanish 2004	Inpatients at the institution, referred to the cardiology consult service for suspected CHF	Patients with non-sinus rhythm, severe mitral regurgitation, mitral stenosis, a prosthetic mitral valve, or severe mitral annular calcification, which reduce the accuracy of Doppler in estimating left ventricular filling pressures. Also patients with unstable angina or myocardial infarction becasue BNP can be elevated in these conditions.
Fischer 2001	Cardiac: With underlying cardiac disease and suspected CHF.<br>Reference: Healthy.	Insufficient samples or clinical data
Fuat 2006	Referrals from general practice of patients with symptoms and signs suggestive of HF	None stated
Gorrisen 2007	Who presented to the emergency department with dyspnoea as their primary complaint	Patients who presented with acute myocardial infarction
Gruson 2009	Admitted to emergency department with dyspnea and/or chest pain	None stated
Jungbauer 2012	Study: Patients with structural heart disease from HF and implantable cardioverter defibrillator	None stated

	outpatient clinic.<br>Controls: Healthy hospital staff	
Knudsen 2004	Admitted to the emergency department with a principal complaint of shortness of breath	Patients with chest pain and patients in whom the shortness of breath was clearly not secondary to CHF (e.g. trauma).
**San Diego Vets**<br>Krishnaswamy 2001<br><br>Lubien 2002	Referred for echocardiography to assess left ventricular function between June and August 2000<br><br>Referred by clinic physician or nurse practitioners for echocardiography to evaluate left ventricular function between June 1999 and November 2000	Patients whose referral was to assess valve disease, to determine whether a vegetation was present, or to rule out a cardiac cause of a stroke.<br><br>Patients whose referral was to assess valve disease, to determine whether a vegetation was present, or to rule out a cardiac cause of stroke. Patients with an ejection fraction <50% or an left ventricular end-diastolic dimension >5.5 mm.
Lainchbury 2003	Presenting to the emergency department with acute dyspnea	If unable to give a blood sample within 8hr of arrival
Logeart 2002	patients presenting to the emergency room for acute severe dyspnea	Patients with acute myocardial infarction, chest injury, recent surgery, those with treatment started more than 2 hours before arrival and those for which echo was not feasible.
Maisel 2001	Referred for echocardiography to evaluate the presence or absence of left ventricular dysfunction	Patients with known left ventricular dysfunction and those referred for echocardiography to assess valve disease, the presence of a vegetation, or to

		rule out a cardiac cause of stroke.
**BNP study**<br>Maisel 2002<br>Maisel 2003<br>Pahle 2009	Aged 18 and over. Arrival at emergency department with shortness of breath as the most prominent symptom.	Patients with unstable angina or whose dyspnea was clearly not secondary to congestive heart failure (for example, those with trauma or cardiac tamponade) and those with acute myocardial infarction or renal failure.
Mak 2004	Referrals for echocardiographic evaluation of cardiac structure and function	Patients whose referral was to assess valvular disease, to determine whether vegetation was present, or to rule out a cardiac cause of stroke. Patients with known significant valvular disease referred for other reasons or found to have significant valvular disease
Monfort 2015	Patients with stable CHF who were followed at the cardiac rehabilitation unit at Lariboisière University Hospital	None stated.
Morrison 2002	Patients presenting to urgent care with dyspnea, with shortness of breath either at rest, with exertion or upon lying down, as a prominent complaint. Other associated symptoms could be edema, weight gain, cough or wheezing.	Patients whose dyspnea was clearly not secondary to CHF (knife wounds, trauma and cardiac tamponade). Patients with unstable angina or acute myocardial infarction were excluded unless their predominant presentation was dyspnea."
Prontera 2005	Study: Aged 18 and over. With idiopathic or secondary cardiomyopathy (heart failure group)<br>Reference: Healthy	None stated.

Prosen 2011	Aged 18 and over. Shortness of breath as the primary complaint (defined as either the sudden onset of dyspnea without history of chronic dyspnea or an increase in the severity of chronic dyspnea)	History of renal insufficiency, trauma, severe coronary ischemia (unless patient's predominant presentation was dyspnea) and other causes of dyspnea, comprising pneumonia, pulmonary embolism, carcinoma, pneumothorax, pleural effusion, intoxication (drugs), anaphylactic reactions, upper airway obstruction, bronchial stenosis , gastroesophageal reflux disorder according to the history, clinical status and additional lab tests available in the pre-hospital setting (D-dimer, troponin, C-reactive protein)
Ro 2011	Aged 18 and over. Patients who presented to the emergency department with symptoms suggestive of heart failure that resulted in a BNP test being ordered as part of their standard of care, and provided signed written consent.	Patients who were unwilling or unable to provide written consent, were already a hospital inpatient, were participating in any study that was deemed by the investigator to confound the intended results of the study, or were considered a vulnerable population for the study by the investigator
Shao 2005	Patients with dyspnea	External injury, acute coronary syndrome, trauma, pericardial tamponade.
Storti 2004	Cardiac and confirmed HF groups: Admitted to cardiovascular medical and surgical departments of hospital from Jan to April 2004<br>Reference: Healthy	None stated.

Tang 2005	Study: Patients with a known diagnosis of heart failure and varying symptom severity (New York Heart Association [NYHA] class I-IV).<br>Controls: Healthy	Any individual receiving intravenous Nesiritide infusions or with significant renal insufficiency.
Taylor 2017	Primary care patients aged >55 years presenting with recent new-onset shortness of breath, lethargy, or peripheral ankle oedema of >48 hours' duration for which there was no other obvious cause.	If unable to give consent, had a previous confirmed diagnosis (that is, with objective evidence) of heart failure, an obvious alternative diagnosis, severe symptoms requiring immediate management, or recent (within 60 days) acute coronary syndrome.
Tomonaga 2011	Presenting with potentially cardiovascular chest pain or symptoms	Refusal of consent, presentation >5 days after symptom onset, recent anticoagulant treatment, severe renal dysfunction and cancer therapy.
Vanderheyden 2006	Patients with suspected heart disease, referred for elective diagnostic heart catheterization	Patients with atrial fibrillation, unstable angina, recent myocardial infarction, severe liver disease, renal insufficiency defined by a serum creatinine >176.8 µmol/L and/or estimated glomerular filtration rate (eGFR) < 60 mL/min
Verdu 2012	Patients for whom echocardiography was requested by a primary care physician to investigate suspected heart failure.	Patients with a previous diagnosis of HF or severe valve disease in the digitized clinical history, those included in a home care program, and those who did not give their consent to participate in the study.
Villacorta 2002	Presenting to the emergency department with acute dyspnea	Patients with a clear diagnosis of dyspnea, such as trachea stenosis or cardiac tamponade, were

		excluded as were those with acute coronary syndromes whose prominent complaint was not dyspnea.
Watson 2016	With cardiovascular risk factors, recruited from primary care practices in Ireland. Age 40 years and over and at least one risk factor for left ventriculardysfunction including: type 2 diabetes, hypertension, obesity, hypercholesterolemia, or coronary artery disease. DM was defined as fasting plasma glucose 7.0 mmol/L or 2 h postload plasma glucose of 11.1 mmol/L.	Those who refused to provide informed consent, had established evidence of left ventricular systolic dysfunction, had evidence or a history of symptomatic heart failure, or had a diagnosis compromising survival over the study period of the trial.
Weekes 2016	With confirmed acute pulmonary embolism according to the results of computed tomography angiography or high probability ventilation-perfusion scan. Over 18 years.	If massive pulmonary embolism criteria were met, if they were unable to tolerate the echocardiogram, or if the ultrasonographic images were of inadequate quality.
Wei 2005	Hypertensive men.	Chest trauma, pericardial effusion, angina or renal dysfunction<br>prior to the study.
Wieczorek 2002	Study: outpatients and inpatients<br>Controls: Healthy	Patients with end-stage kidney disease, acute myocardial infarction
Zapata 2014	Patients under mechanical ventilation, non-cardiac critically ill.	Declined to participate, admitted to intensive care unit for acute HF, acute myocardial infarction or cardiac surgery, left ventricular ejection fraction<50%, left ventricular end diastolic

		dimension >5.5cm
Zhao 2008	In-hospital patients, all of whom presented shortness of breath and/or dyspnoea as the most prominent symptom.	Valvular heart disease, acute coronary syndromes, pneumothorax, lung cancer, cardiac tamponade, and liver or renal failure.

CHF – congestive/chronic heart failure

Table S2. Results of pooling data from cross-sectional/cohort/randomised control trial studies in ambulatory settings and in populations with a low prevalence of heart failure

Thresholds	No of studies	No of studies in primary care	Sensitivity	Specificity
**BNP**				
30-40 pg/mL	3	2	0.83 (0.72 to 0.91)	0.69 (0.38 to 0.89)
50 pg/mL	5	1	0.93 (0.64 to 0.99)	0.62 (0.41 to 0.79)
100-104 pg/mL	8	0	0.95 (0.91 to 0.98)	0.62 (0.46 to 0.75)
100 pg/mL	7	0	0.95 (0.90 to 0.98)	0.64 (0.46 to 0.78)
150 pg/mL	4	0	0.88 (0.81 to 0.92)	0.80 (0.57 to 0.93)
200-208 pg/mL	4	0	0.88 (0.82 to 0.92)	0.73 (0.62 to 0.81)
300 pg/mL	1	0	0.73 (0.70 to 0.76)	0.89 (0.87 to 0.91)
400 pg/mL	1	0	0.63 (0.59 to 0.67)	0.91 (0.89 to 0.93)
**NT-proBNP**				
125 pg/mL	3	3	0.99 (0.57 to 1.00)	0.60 (0.44 to 0.74)
400 pg/mL	2	2	0.76 (0.49 to 0.91)	0.85 (0.75 to 0.92)

Figure S1. Testing for Publication Bias (Deeks 2005)

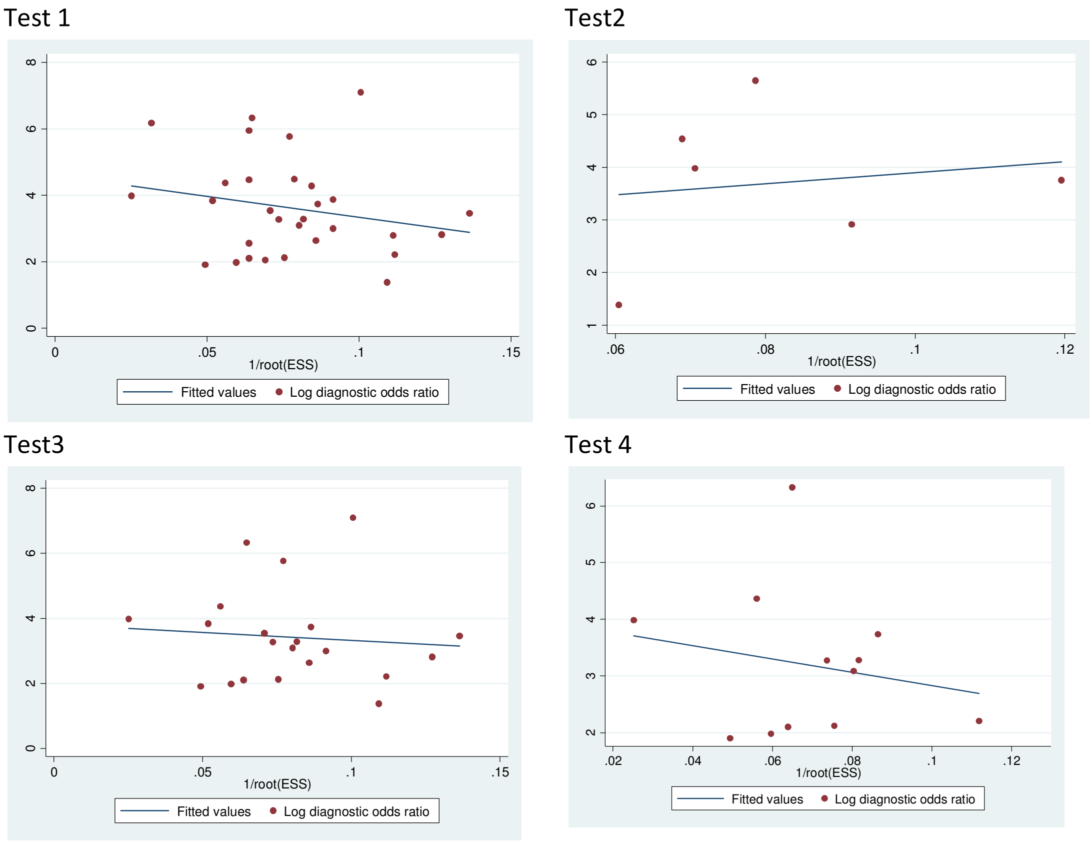

Using data for the lowest threshold for each study to ensure independence assumption was met. Tests: 1- All BNP studies ($p=0.1$); 2 - All NT-proBNP studies ($p=0.5$); 3 - Triage index test, clinical assessment reference test, cross sectional/cohort design, all settings ($p=0.5$); 4 - Triage index test, clinical assessment reference test, cross sectional/cohort design, ambulatory settings, low prevalence of heart failure ($p=0.3$)

Figure S2. Paired forest plots, at two threshold levels, for B type natriuretic peptide compared with clinical assessment, for cross-sectional/cohort studies with populations of low prevalence of heart failure in mixed outpatient and inpatient settings

B type natriuretic peptide below 100 pg/mL

Study	TP	FP	FN	TN	Threshold	Sensitivity (95% CI)	Specificity (95% CI)
Maisel 2001a	90	36	5	69	38.5	0.95 [0.88, 0.98]	0.66 [0.56, 0.75]
Maisel 2001b	88	21	7	84	46.0	0.93 [0.85, 0.97]	0.80 [0.71, 0.87]
Maisel 2001c	87	15	8	90	55.0	0.92 [0.84, 0.96]	0.86 [0.78, 0.92]
Maisel 2001d	84	9	11	96	65.0	0.88 [0.80, 0.94]	0.91 [0.84, 0.96]
Maisel 2001e	82	2	13	103	75.0	0.86 [0.78, 0.93]	0.98 [0.93, 1.00]
Mak 2004a	15	27	1	57	90.0	0.94 [0.70, 1.00]	0.68 [0.57, 0.78]

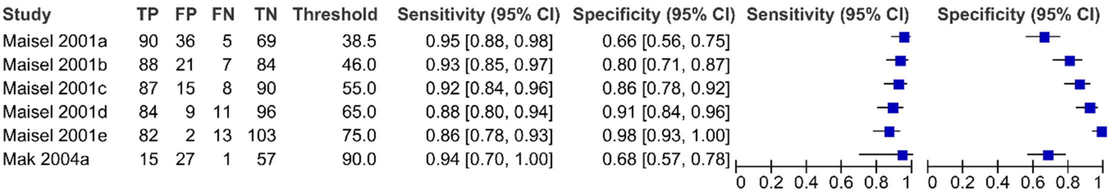

B type natriuretic peptide at and above 100 pg/mL

Study	TP	FP	FN	TN	Threshold	Sensitivity (95% CI)	Specificity (95% CI)
Mak 2004b	14	15	2	69	173.0	0.88 [0.62, 0.98]	0.82 [0.72, 0.90]
Mak 2004c	12	9	4	75	279.0	0.75 [0.48, 0.93]	0.89 [0.81, 0.95]
Mak 2004d	8	6	8	78	402.0	0.50 [0.25, 0.75]	0.93 [0.85, 0.97]

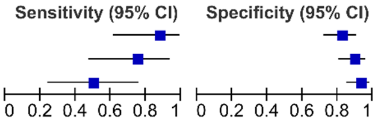

Based on data for 2 studies. All index tests were Triage. TP – true positives; FP – false positives; FN – false negatives; TN – true negatives

Figure S3. Paired forest plots for N-Terminal pro-B type natriuretic peptide compared with clinical assessment, at two threshold levels for case-control studies

NT Pro-BNP <125 pg/mL

Study	TP	FP	FN	TN	Threshold	Sensitivity (95% CI)	Specificity (95% CI)
Ajuluchukwu 2009a	39	7	3	23	95.0	0.93 [0.81, 0.99]	0.77 [0.58, 0.90]

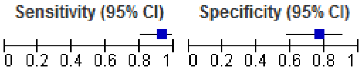

NT-proBNP below 400 pg/mL

Study	TP	FP	FN	TN	Threshold	Sensitivity (95% CI)	Specificity (95% CI)
Ajuluchukwu 2009a	39	7	3	23	95.0	0.93 [0.81, 0.99]	0.77 [0.58, 0.90]
Ajuluchukwu 2009b	39	6	3	24	100.0	0.93 [0.81, 0.99]	0.80 [0.61, 0.92]
Ajuluchukwu 2009c	39	5	3	25	105.0	0.93 [0.81, 0.99]	0.83 [0.65, 0.94]
Ajuluchukwu 2009d	39	5	3	25	110.0	0.93 [0.81, 0.99]	0.83 [0.65, 0.94]
Ajuluchukwu 2009e	39	5	3	25	113.0	0.93 [0.81, 0.99]	0.83 [0.65, 0.94]
Ajuluchukwu 2009f	38	5	4	25	115.0	0.90 [0.77, 0.97]	0.83 [0.65, 0.94]
Ajuluchukwu 2009g	37	5	5	25	120.0	0.88 [0.74, 0.96]	0.83 [0.65, 0.94]
Ajuluchukwu 2009h	37	5	5	25	122.0	0.88 [0.74, 0.96]	0.83 [0.65, 0.94]
Ajuluchukwu 2009i	37	5	5	25	123.0	0.88 [0.74, 0.96]	0.83 [0.65, 0.94]
Ajuluchukwu 2009j	37	4	5	26	124.0	0.88 [0.74, 0.96]	0.87 [0.69, 0.96]
Ajuluchukwu 2009k	37	4	5	26	125.0	0.88 [0.74, 0.96]	0.87 [0.69, 0.96]
Ajuluchukwu 2009l	36	4	6	26	126.0	0.86 [0.71, 0.95]	0.87 [0.69, 0.96]
Ajuluchukwu 2009m	36	4	6	26	127.0	0.86 [0.71, 0.95]	0.87 [0.69, 0.96]
Ajuluchukwu 2009n	36	4	6	26	130.0	0.86 [0.71, 0.95]	0.87 [0.69, 0.96]
Ajuluchukwu 2009o	36	4	6	26	135.0	0.86 [0.71, 0.95]	0.87 [0.69, 0.96]
Ajuluchukwu 2009p	36	4	6	26	140.0	0.86 [0.71, 0.95]	0.87 [0.69, 0.96]
Ajuluchukwu 2009q	36	4	6	26	145.0	0.86 [0.71, 0.95]	0.87 [0.69, 0.96]
Ajuluchukwu 2009r	36	2	6	28	171.0	0.86 [0.71, 0.95]	0.93 [0.78, 0.99]

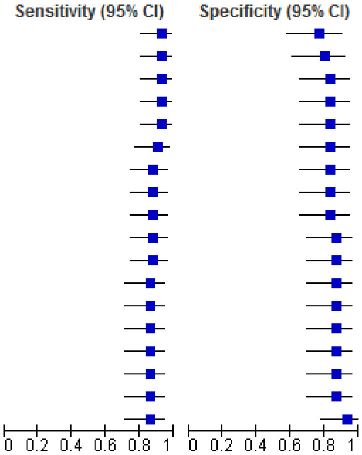

NT-proBNP at and above 400 pg/mL

Study	TP	FP	FN	TN	Threshold	Sensitivity (95% CI)	Specificity (95% CI)
Jungbauer 2012b	49	7	4	162	410.0	0.92 [0.82, 0.98]	0.96 [0.92, 0.98]

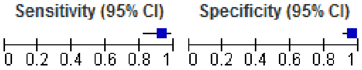

Based on data for 2 studies. All index tests were Cardiac Reader. Reference tests were clinical assessment based on single or multiple tests. TP – true positives; FP – false positives; FN – false negatives; TN – true negatives

Figure S4. Sensitivity analysis: Summary Receiver-Operating-Characteristics plots for B type natriuretic peptide and N-Terminal pro-B type natriuretic peptide, compared with clinical assessment, for cross-sectional/cohort/randomised controlled trial studies of populations in ambulatory settings, selecting the lowest threshold for each study.

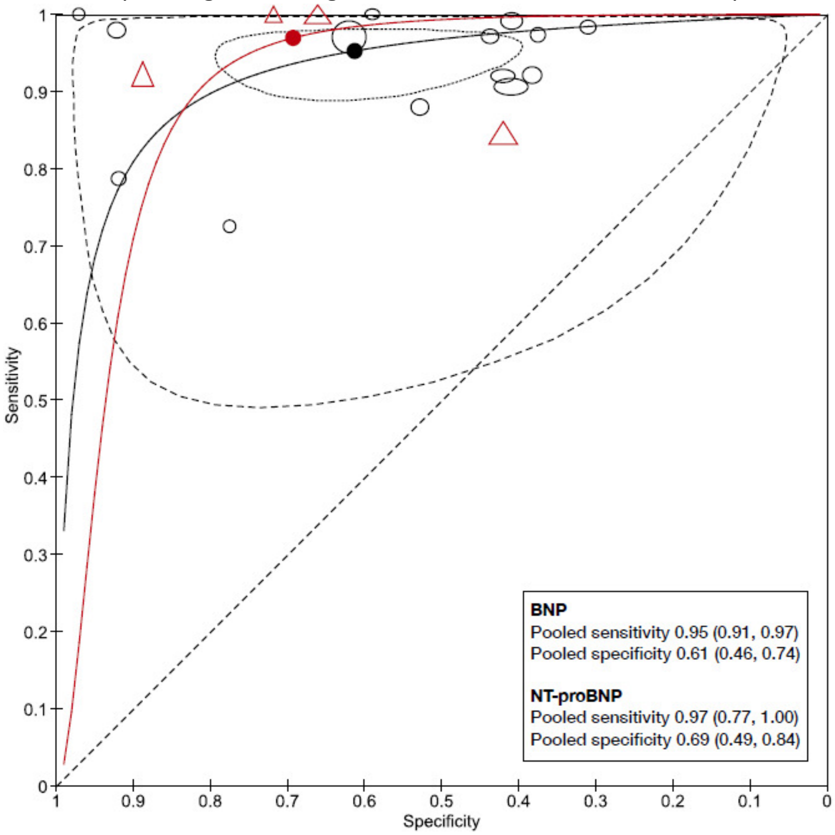

Based on data for 17 studies; 4 NT-proBNP and 13 BNP studies. Size of symbol indicates study size. There were insufficient studies to draw meaningful prediction and confidence regions for NT-proBNP. BNP - Triage, black circle; NT-proBNP - Cardiac Reader, red triangle.

APPENDICES

Appendix 1. Research protocol

Systematic review: Accuracy of point-of-care B-type natriuretic peptide testing in the diagnosis and monitoring of chronic heart failure

Introduction

Target condition:

An estimated 800,000 people in the UK currently have heart failure with an estimate of over 250 000 new cases of heart failure in the UK every year. Incidence increases with age and is highest in adults over 75 [1]. The ageing population and improved survival of individuals with ischaemic heart disease are likely to lead to a continuing rise in the prevalence of heart failure. Overall a general practitioner (GP) with a patient population of 2000 will care for approximately 40-50 patients with heart failure and see 2-3 new cases each year.

Since heart failure may be reversible in the early stages it is important that heart failure is diagnosed as quickly as possible. However, because it has a low incidence, GPs are unlikely to have sufficient experience to identify more subtle presentations of heart failure. For example, whilst heart failure is frequently diagnosed by GPs, it is only confirmed by echocardiography in approximately a third of cases [2]. A recent health technology assessment of the use of B-type natriuretic peptide (BNP) in the diagnosis of heart failure, compared with ECG and echocardiography found that a normal ECG can be used to exclude a diagnosis of heart failure but it is relatively non-specific. Whilst ECG is a sensitive test when performed by cardiologists (sensitivity = 89%; 95% CI 77%–95%) [3], its sensitivity was much lower (53%) when carried out by GPs [4]. It is for this reason that the most recent NICE guidelines on chronic heart failure (CHF) have refined the diagnostic algorithm for heart failure, replacing ECG with serum BNP measurement [5]. POC BNP testing would enable GPs to rapidly refer the appropriate patients or, if CHF can be excluded, investigate alternative causes of dyspnoea.

Recent National Institute for Health and Clinical Excellence (NICE) and European Society for Cardiology (ESC) guidelines on the initial diagnosis of CHF and referral for echocardiography recommend the use of BNP in combination with clinical assessment [5, 6]. NICE guidelines recommend measurement of serum natriuretic peptides in patients with suspected heart failure

without previous myocardial infarction (MI), and those with previous MI should be referred for an urgent echocardiogram [5]. Timely diagnosis and treatment of CHF may result in improved patient outcomes as has been demonstrated for acute decompensated heart failure (ADHF) [7]. Although several hospital laboratories carry out BNP testing, few return results within a day. Point-of-care (POC) BNP testing can considerably reduce turnaround time and could lead to earlier initial treatment, more timely referral and less uncertainty for patients. Moreover, using POC BNP levels to quickly rule out heart failure, could allow more rapid initiation of investigation of other causes of dyspnoea.

Index test:

B-type natriuretic peptide is produced from heart muscle cells as a pro-hormone (proBNP) and released into the cardiovascular system in response to ventricular dilation and pressure overload [8]. The pro-hormone is split by a protease and secreted as the physiologically active C-terminal fragment (BNP) and the inactive N-terminal fragment (NT-proBNP). Several POC BNP testing devices are on the market, which either measure BNP or NT-proBNP (the latter has a longer half-life), for example:

BNP:

1. Biosite Triage System BNP Test (Biosite Diagnostics Inc., San Diego, CA; Supplied in the UK by Alere, Stockport [previously Inverness Medical]). This immunoassay that measures BNP concentration from capillary whole blood samples obtained by finger prick is CE marked and CLIA waived for use with whole blood. Results are available in 12-15 minutes. Information provided by the manufacturer reports the device has lower and upper detection limits of 5 pg/ml and 5000 pg/ml, respectively (http://www.alere.com/us/en/product-details/triage-bnp-test.html). This is a desk-top device which weighs approximately 0.7kg and is portable. Initial studies claim the test has a 98% diagnostic accuracy at a cut-off value of 80 pg/ml in an urgent care setting [9].
2. Alere Heart Check System (Alere, Stockport, UK; CE marked). This test measures BNP concentration from a 15µl sample (obtained by finger prick) and results are available in 15 minutes. The product is handheld. (http://www.alereheartcheck.com/content/alere-heart-check/home.html)
3. Abbott iSTAT Analyser (Abbott Point of Care, IL, USA; Supplied in the UK by Axis-Shield; CE marked). This test measures BNP concentration from a 17µl sample (obtained by finger prick) and results are available in 10 minutes. The device has reported lower and upper

detection limits of 15 pg/ml and 5000 pg/ml, respectively (http://www.abbottpointofcare.com/). The product is handheld.

NT-proBNP:

1. RAMP 200 Clinical System (Response Biomedical, BC, Canada; no UK distributor identified; CE marked). This test measures NT-proBNP from an EDTA whole blood sample, results are available in 15 minutes. The device has a reported lower limit of detection of18 pg/ml and an upper limit of linearity of 23,450 pg/ml [10]. The product weighs approximately 2 kg and is portable.
2. Cobas h 232/Cardiac Reader (Roche Diagnostics, Burgess Hill, UK; CE marked). This test measures NT-proBNP from a 150µl sample of heparinised venous blood; results available in 12 minutes. The device has reported lower and upper limits of detection of 60 pg/ml and 3000 pg/ml, respectively (https://www.cobas-roche.co.uk). The product is handheld.

Importantly, BNP levels have been found to vary with age, gender and certain diseases (e.g. renal failure) [8]. It has been suggested that higher cut-off values are used when individuals are >75 years, female or in renal failure.

Reference standards:

In the literature point-of-care BNP tests are compared to laboratory-based systems (e.g. Roche Elecsys analyser, Abbott ARCHITECT) and echocardiography and/or clinical examination by cardiologists is frequently used as the reference standard [2-4, 10-15].

Rationale

Systematic reviews have been compiled on the role and accuracy of BNP and NT-proBNP in the diagnosis of chronic heart failure [3, 12, 13, 15], however none have focussed specifically on the accuracy of point-of-care testing, particularly in the primary care setting. As mentioned above, several devices are currently on the market, however a systematic review on the evidence for the accuracy of the devices is currently lacking.

Clinical question

In patients presenting to primary care with suspected or confirmed chronic heart failure, what is the accuracy of point-of-care (POC) BNP devices compared to a reference standard (including laboratory-based tests, echocardiography or clinical examination or combinations of these) for the diagnosis and monitoring of chronic heart failure.

Therefore:

Population: patients with suspected chronic heart failure, or patients with chronic heart failure currently being monitored. The setting of particular interest is Primary Care, but patients from other settings (hospital, specialist clinic) will also be included

Index test: POC/near patient/bedside BNP tests

Reference standard: laboratory-based BNP tests or echocardiography or clinical examination or combinations of these.

Target condition: chronic heart failure

Objective

Compiling a systematic review and meta-analysis (if appropriate) of studies that investigate the diagnostic accuracy of POC BNP tests in patients with dyspnoea/suspected heart failure or with a diagnosis of heart failure. While the review will include studies from any setting, we will also seek to summarise the evidence in primary care, if possible.

Methods and Data Collection:

Inclusion criteria:

1. Study designs: To include cross-sectional, longitudinal and case-control designs; both prospective and retrospective. Randomised controlled trials, cohort and case-control studies will be included.
2. Index test and Reference standard: Studies comparing POC-BNP and/or POC NT-proBNP tests with any reference standard (including but not limited to laboratory-based tests, echocardiography or clinical examination or combinations of these). Studies comparing different POC tests with each other may also be considered, but will need to include a comparison with a laboratory or clinical reference standard. The index test will be any portable test that can be performed at the point of care that does not require laboratory services. Large bench-top devices will be excluded.
3. Outcomes: Studies reporting accuracy measures, such as sensitivity, specificity, likelihood ratios and predictive values, Bland-Altman plots etc. will be included.
4. Participants and setting: Studies with participants with suspected or confirmed Chronic Heart Failure of any age in all settings will be included. No restrictions will be made regarding population numbers.
5. Study quality will not be a basis for exclusion.
6. Language: Any language.

Exclusion criteria:

1. Not in humans/ using non-human samples
2. Not a primary study
3. Does not include point-of-care BNP or point-of-care NT-proBNP testing
4. No reference standard or comparator
5. Diagnostic accuracy not reported as an outcome
6. Data not provided to allow for construction of 2x2 table

Search Strategy:

The search strategy will be developed in consultation with a healthcare librarian experienced with supporting systematic reviews. Multiple electronic databases will be searched including, but not confined to, MEDLINE, EMBASE, the Cochrane library, TRIP database and the Science Citation Index. Free text and MeSH headings will be used. The search may use relevant filters, but in order to maximise sensitivity, will not be limited to these. The reference lists of relevant studies will be examined and additional tools such as the "related articles" feature in PubMed will also be used to identify relevant publications. Clinical trials registries will be searched and manufacturers of POC BNP/NT-proBNP devices will be contacted for information on any unpublished studies.

Study Selection:

Search results will be screened by two reviewers independently. Full text of relevant studies will be obtained and assessed for inclusion by each reviewer. Disagreements will be resolved by discussion or referral to a third reviewer. Study identification will be summarised in a PRISMA flow diagram.

Data extraction:

A data extraction form will be developed and data extraction will be performed independently by 2 reviewers, cross-checked and disagreements will be resolved by discussion or referral to a third reviewer.

Extracted data will include:

1. *Study identification*- author, year, and location.
2. *Study details*- cohort, case-control, randomised trial. To include cross-sectional, longitudinal and case-control designs; both prospective and retrospective.
3. *Participants or samples:* to include number, and age, gender, condition(s), illness severity, etc.
4. *Setting:* primary care, out-of-hours, emergency care, hospital or clinic setting
5. *Index test* – details of POC test used (e.g. manufacturer, type, etc.) and user (e.g. clinician, nurse, laboratory worker, patient etc.)

6. *Comparator or reference test* – details of nature of reference test (e.g. laboratory, clinical examination, ECG, etc.)
7. *Outcome measures* – diagnostic accuracy measures (e.g. sensitivity, specificity, likelihood ratios, predictive values, etc.) and primary data for 2x2 tables.

Quality assessment:

QUADAS2 (the Revised Tool for Quality Assessment of Diagnostic Accuracy Studies; [16]) will be used to assess the study quality of each included study and will be reported in table or graphics form.

Data analysis:

Summary Tables

Summary tables will detail the patient sample, study design, the test under evaluation, the thresholds at which accuracy has been reported, and the comparator.

Meta-analysis

For each test, RevMan will be used to produce paired forest plots to explore the between-study variability of sensitivity and specificity across the included studies. For each study estimate of sensitivity and specificity, corresponding 95% confidence intervals will be shown to illustrate the uncertainty related to each study estimate. If accuracy has been reported at multiple common thresholds, forest plots will be sub-grouped on threshold.

Bivariate meta-analysis methods will be used to generate pooled estimates of sensitivity and specificity where sufficient data is available for each test. Hierarchical summary ROC meta-analysis methods will be used to produce summary ROC curves with corresponding 95% confidence region and prediction region. We will use the xtmelogit command in Stata for these analyses and feed parameters directly into Revman to produce Cochrane-standardised output.

Investigations of heterogeneity

Two approaches will be used to explore the sources of between-study heterogeneity: 1) adjusting for possible sources by adding them as covariates to the bivariate model 2) carrying out sub-group analyses (e.g. subgroup analysis excluding studies using a case-control design, which is known to introduce bias). The latter will only be carried out if there is sufficient data available and sub-group specific pooled estimates are thought to be of clinical relevance.

Sensitivity analyses

If there appear to be any outliers in the data, these studies will be removed from the analysis to evaluate the impact on the overall pooled estimates.

Assessment of reporting bias

Funnel plots used to detect publication bias in reviews of RCTs have also been shown to be misleading for DTA reviews [17-19]. Assessment of reporting bias will therefore not be included in this review.

References

1. Townsend, N., et al., *Coronary heart disease statistics 2012 edition*, 2012, British Heart Foundation: London.
2. Zaphiriou, A., et al., *The diagnostic accuracy of plasma BNP and NTproBNP in patients referred from primary care with suspected heart failure: results of the UK natriuretic peptide study.* Eur J Heart Fail, 2005. **7**(4): p. 537-41.
3. Mant, J., et al., *Systematic review and individual patient data meta-analysis of diagnosis of heart failure, with modelling of implications of different diagnostic strategies in primary care.* Health Technol Assess, 2009. **13**(32): p. 1-207, iii.
4. Zuber, M., et al., *Value of brain natriuretic peptides in primary care patients with the clinical diagnosis of chronic heart failure.* Scand Cardiovasc J, 2009. **43**(5): p. 324-9.
5. *National Institute for Health and Clinical Excellence Guideline 108: Chronic heart failure national clinical guideline for diagnosis and management in primary and secondary care*, 2010.
6. Dickstein, K., et al., *ESC guidelines for the diagnosis and treatment of acute and chronic heart failure 2008: the Task Force for the diagnosis and treatment of acute and chronic heart failure 2008 of the European Society of Cardiology. Developed in collaboration with the Heart Failure Association of the ESC (HFA) and endorsed by the European Society of Intensive Care Medicine (ESICM).* Eur J Heart Fail, 2008. **10**(10): p. 933-89.
7. Peacock, W.F.t., et al., *Impact of early initiation of intravenous therapy for acute decompensated heart failure on outcomes in ADHERE.* Cardiology, 2007. **107**(1): p. 44-51.
8. Balion, C., et al., *Testing for BNP and NT-proBNP in the diagnosis and prognosis of heart failure.* Evid Rep Technol Assess (Full Rep), 2006(142): p. 1-147.
9. Dao, Q., et al., *Utility of B-type natriuretic peptide in the diagnosis of congestive heart failure in an urgent-care setting.* J Am Coll Cardiol, 2001. **37**(2): p. 379-85.
10. Lee-Lewandrowski, E., et al., *Multi-center validation of the Response Biomedical Corporation RAMP NT-proBNP assay with comparison to the Roche Diagnostics GmbH Elecsys proBNP assay.* Clin Chim Acta, 2007. **386**(1-2): p. 20-4.

11. Alehagen, U. and M. Janzon, *A clinician's experience of using the Cardiac Reader NT-proBNP point-of-care assay in a clinical setting.* Eur J Heart Fail, 2008. **10**(3): p. 260-6.
12. Korenstein, D., et al., *The utility of B-type natriuretic peptide in the diagnosis of heart failure in the emergency department: a systematic review.* BMC Emerg Med, 2007. **7**: p. 6.
13. Latour-Perez, J., et al., *Accuracy of B-type natriuretic peptide levels in the diagnosis of left ventricular dysfunction and heart failure: a systematic review.* Eur J Heart Fail, 2006. **8**(4): p. 390-9.
14. Shah, K., et al., *Comparability of Results between Point-of-Care and Automated Instruments to Measure B-type Natriuretic Peptide.* West J Emerg Med, 2010. **11**(1): p. 44-8.
15. Wang, C.S., et al., *Does this dyspneic patient in the emergency department have congestive heart failure?* JAMA, 2005. **294**(15): p. 1944-56.
16. Whiting, P.F., et al., *QUADAS-2: a revised tool for the quality assessment of diagnostic accuracy studies.* Ann Intern Med, 2011. **155**(8): p. 529-36.
17. Deeks, J.J., P. Macaskill, and L. Irwig, *The performance of tests of publication bias and other sample size effects in systematic reviews of diagnostic test accuracy was assessed.* J Clin Epidemiol, 2005. **58**(9): p. 882-93.
18. Leeflang, M.M., et al., *Systematic reviews of diagnostic test accuracy.* Ann Intern Med, 2008. **149**(12): p. 889-97.
19. Song, F., et al., *Asymmetric funnel plots and publication bias in meta-analyses of diagnostic accuracy.* Int J Epidemiol, 2002. **31**(1): p. 88-95.

Appendix 2. MEDLINE search terms

**#**	**Searches**
1	exp Heart Failure/
2	(heart adj5 fail*).ti,ab.
3	(cardiac adj5 (fail* or insufficien*)).ti,ab.
4	(chf or hf).ti,ab.
5	1 or 2 or 3 or 4
6	exp natriuretic peptides/ or natriuretic peptide, brain/
7	((brain or btype or b type) adj3 natriuretic peptide*).ti,ab.
8	bnp.ti,ab.
9	(ntprobnp or ntpro bnp or nt pro bnp).ti,ab.
10	(probnp or pro bnp).ti,ab.

11	((n terminal or ntpro or nt pro) adj5 natriuretic peptide*).ti,ab.
12	6 or 7 or 8 or 9 or 10 or 11
13	((immediate$ or rapid$ or same time or same visit or near patient or instant$ or portable or handheld or hand-held or bedside or bed-side) adj3 (test$ or turnaround or analys$ or analyz$ or measure$ or assay$ or monitor* or device*)).tw.
14	(fingerprick or finger prick).tw.
15	(poc or poct or "point of care").tw.
16	Point-of-Care Systems/
17	13 or 14 or 15 or 16
18	5 and 12 and 17
19	((immediate$ or rapid$ or same time or same visit or near patient or instant$ or portable or handheld or hand-held or bedside or bed-side) adj5 ((brain or btype or b type) adj3 natriuretic peptide*)).ti,ab.
20	((immediate$ or rapid$ or same time or same visit or near patient or instant$ or portable or handheld or hand-held or bedside or bed-side) adj5 bnp).ti,ab.
21	((immediate$ or rapid$ or same time or same visit or near patient or instant$ or portable or handheld or hand-held or bedside or bed-side) adj5 (probnp or pro bnp)).ti,ab.
22	((immediate$ or rapid$ or same time or same visit or near patient or instant$ or portable or handheld or hand-held or bedside or bed-side) adj5 (ntprobnp or ntpro bnp or nt pro bnp)).ti,ab.
23	((immediate$ or rapid$ or same time or same visit or near patient or instant$ or portable or handheld or hand-held or bedside or bed-side) adj5 ((n terminal or ntpro or nt pro) adj5 natriuretic peptide*)).ti,ab.
24	(biosite triage or alere heart check or heart check system or abbott istat or ramp 200 or cobas h 232 or cardiac reader).ti,ab.
25	19 or 20 or 21 or 22 or 23 or 24
26	5 and 25
27	18 or 26
28	exp animals/ not humans.sh.
29	27 not 28

Appendix 3. Variables in data extraction sheet

To evaluate eligibility: First author; study reference; title; journal details; and, eligibility criteria
To record study design: First author; study reference; study aim; design; whether data was collected prospectively, retrospectively or unclear; consecutive recruitment or not; population or samples; inclusion criteria; age and gender data for study population and control population (if relevant); setting; country; cause of heart failure; interval between index and reference test; details about each index test (name/company/type, what is measured, sample type, threshold used, whether threshold was pre-specified, whether or not all patients were tested); details about each reference test (name/company/type, what is measured, sample type, threshold used, whether threshold was pre-specified, whether or not all patients were tested, whether or not they received the same reference test); target condition; primary and secondary outcomes; funding source.
To evaluate methodological quality: All QUADAS-2 variables
To record the diagnostic accuracy results: First author; study reference; total number of patients; number of patients with heart failure; name of index test; name of reference test; what is measured (BNP or NT-proBNP); statistical measure; patient subgroup (if relevant); target condition; threshold; units; value; lower confidence interval; upper confidence interval.
Authors' analysis: Basic conclusions of study; our general comments

Appendix 4. References of studies that satisfied eligibility criteria, listed alphabetically

Ajuluchukwu J, Ekure E, Mbakwem A. et al. Reliability and accuracy of point-of-care amino-terminal probrain natriuretic peptide in congestive heart failure patients. *Internet J Cardiol*. 2009;9(2)

Alibay Y, Beauchet A, El Mahmoud R, et al. Plasma N-terminal pro-brain natriuretic peptide and brain natriuretic peptide in assessment of acute dyspnea. *Biomed Pharmacother*. 2005 Jan-Feb;59(1-2):20-4. Epub 2005 Jan 20.

Blondé-Cynober F, Morineau G, Estrugo B, et al. Diagnostic and prognostic value of brain natriuretic peptide (BNP) concentrations in very elderly heart disease patients: specific geriatric cut-off and impacts of age, gender, renal dysfunction, and nutritional status. *Arch Gerontol Geriatr*. 2011 Jan-Feb;52(1):106-10. doi: 10.1016/j.archger.2010.02.010. Epub 2010 Mar 11.

Breathing Not Properly (BNP) study

Maisel AS, Krishnaswamy P, Nowak RM, et al. Breathing Not Properly Multinational Study Investigators. Rapid measurement of B-type natriuretic peptide in the emergency diagnosis of heart failure. *N Engl J Med.* 2002 Jul 18;347(3):161-7.

Maisel AS, McCord J, Nowak RM, et al Breathing Not Properly Multinational Study Investigators.

Bedside B-Type natriuretic peptide in the emergency diagnosis of heart failure with reduced or preserved ejection fraction. Results from the Breathing Not Properly Multinational Study. *J Am Coll Cardiol.* 2003 Jun 4;41(11):2010-7.

Pahle AS, Sørli D, Omland T, et al. Impact of systemic hypertension on the diagnostic performance of B-type natriuretic peptide in patients with acute dyspnea. *Am J Cardiol*. 2009 Oct 1;104(7):966-71. doi: 10.1016/j.amjcard.2009.05.039.

Chenevier-Gobeaux C, Guerin S, André S, et al. doi: 10.1373/clinchem.2010.145417. Epub 2010 Sep 2. Midregional pro-atrial natriuretic peptide for the diagnosis of cardiac-related dyspnea according to renal function in the emergency department: a comparison with B-type natriuretic peptide (BNP) and N-terminal proBNP. *Clin Chem*. 2010 Nov;56(11):1708-17.

Dao Q, Krishnaswamy P, Kazanegra R, et al. Utility of B-type natriuretic peptide in the diagnosis of congestive heart failure in an urgent-care setting. *J Am Coll Cardiol*. 2001 Feb;37(2):379-85.

De Vecchis R, Ariano C. 2016. Measuring B-Type Natriuretic Peptide From Capillary Blood or Venous Sample: Is It the Same? *Cardiol Res* 7: 51-8.

Dokainish H, Zoghbi WA, Lakkis NM, et al. Comparative accuracy of B-type natriuretic peptide and tissue Doppler echocardiography in the diagnosis of congestive heart failure. *Am J Cardiol*. 2004 May 1;93(9):1130-5.

Fischer Y, Filzmaier K, Stiegler H, et al. Evaluation of a new, rapid bedside test for quantitative determination of B-type natriuretic peptide. *Clin Chem*. 2001 Mar;47(3):591-4.

Fuat A, Murphy JJ, Hungin AP, et al. 2006. The diagnostic accuracy and utility of a B-type natriuretic peptide test in a community population of patients with suspected heart failure. *Br J Gen Pract* 56: 327-33

Gorissen C, Baumgarten R, de Groot M, et al. Analytical and clinical performance of three natriuretic peptide tests in the emergency room*. Clin Chem Lab Med*. 2007;45(5):678-84.

Gruson D, Thys F, Ketelslegers JM, et al. Multimarker panel in patients admitted to emergency department: a comparison with reference methods. *Clin Biochem*. 2009 Feb;42(3):185-8. doi: 10.1016/j.clinbiochem.2008.08.078. Epub 2008 Sep 3.

Jungbauer CG, Kaess B, Buchner S, et al. Equal performance of novel N-terminal proBNP (Cardiac proBNP®) and established BNP (Triage BNP®) point-of-care tests. *Biomark* Med. 2012 Dec;6(6):789-96. doi: 10.2217/bmm.12.67.

Knudsen CW, Riis JS, Finsen AV, et al. Diagnostic value of a rapid test for B-type natriuretic peptide in patients presenting with acute dyspnoe: effect of age and gender. *Eur J Heart Fail*. 2004 Jan;6(1):55-62.

Lainchbury JG, Campbell E, Frampton CM, et al. Brain natriuretic peptide and n-terminal brain natriuretic peptide in the diagnosis of heart failure in patients with acute shortness of breath. *J Am Coll Cardiol.* 2003 Aug 20;42(4):728-35.

Logeart D, Saudubray C, Beyne P, et al. Comparative value of Doppler echocardiography and B-type natriuretic peptide assay in the etiologic diagnosis of acute dyspnea. *J Am Coll Cardiol*. 2002 Nov 20;40(10):1794-800.

Maisel AS, Koon J, Krishnaswamy P, et al. Utility of B-natriuretic peptide as a rapid, point-of-care test for screening patients undergoing echocardiography to determine left ventricular dysfunction. *Am Heart J.* 2001 Mar;141(3):367-74.

Mak GS, DeMaria A, Clopton P, Maisel AS. 2004. Utility of B-natriuretic peptide in the evaluation of left ventricular diastolic function: Comparison with tissue Doppler imaging recordings. *Am Heart Jl* 148: 895-902

Monfort A, Da Silva K, Vodovar N, et al. 2015. Clinical evaluation of the Heart Check system, a new quantitative measurement of fresh capillary BNP. *Biomark Med* 9: 1323-30

Morrison LK, Harrison A, Krishnaswamy P, et al. Utility of a rapid B-natriuretic peptide assay in differentiating congestive heart failure from lung disease in patients presenting with dyspnea. *J Am Coll Cardiol.* 2002 Jan 16;39(2):202-9.

Prontera C, Storti S, Emdin M, et al. Comparison of a fully automated immunoassay with a point-of-care testing method for B-type natriuretic peptide. *Clin Chem*. 2005 Jul;51(7):1274-6.

Prosen G, Klemen P, Štrnad M, Grmec S. Combination of lung ultrasound (a comet-tail sign) and N-terminal pro-brain natriuretic peptide in differentiating acute heart failure from chronic obstructive pulmonary disease and asthma as cause of acute dyspnea in prehospital emergency setting. *Crit Care.* 2011;15(2):R114. doi: 10.1186/cc10140. Epub 2011 Apr 14.

Ro R, Thode HC, Jr., Taylor M, Gulla J, Tetrault E, Singer AJ. 2011. Comparison of the diagnostic characteristics of two B-type natriuretic peptide point-of-care devices. *J Emerg Med* 41: 661-7

San Diego Veterans study

Krishnaswamy P, Lubien E, Clopton P, et al. 2001. Utility of B-natriuretic peptide levels in identifying patients with left ventricular systolic or diastolic dysfunction. *Amer J of Med* 111: 274-9

Lubien E, DeMaria A, Krishnaswamy P, et al. Utility of B-natriuretic peptide in detecting diastolic dysfunction: comparison with Doppler velocity recordings. *Circulation*. 2002 Feb 5;105(5):595-601.

Shao LW, Han Y, Wang ZK, Sun J. The diagnostic application of bedside measurement of plasma brain natriuretic in patients with heart failure. *Zhonghua Nei Ke Za Zhi*. 2005 Feb;44(2):99-101. [Article in Chinese]

Storti S, Prontera C, Emdin M, et al. Analytical performance and clinical results of a fully automated MEIA system for brain natriuretic peptide assay: comparison with a point of care testing method. *Clin Chem Lab Med*. 2004;42(10):1178-85.

Su Q, Liu H1, Zhang X1, et al. Diagnostic values of NT-proBNP in acute dyspnea among elderly patients. *Int J Clin Exp Pathol*. 2015 Oct 1;8(10):13471-6. eCollection 2015.

Tang WH, Philip K, Hazen SL, et al. Comparative sensitivities between different plasma B-type natriuretic peptide assays in patients with minimally symptomatic heart failure. *Clin Cornerstone*. 2005;7 Suppl 1:S18-24.

Taylor CJ, Roalfe AK, Iles R, et al. 2017. Primary care REFerral for EchocaRdiogram (REFER) in heart failure: a diagnostic accuracy study. *Br J Gen Pract* 67: e94-e102

Tomonaga Y, Gutzwiller F, Luscher TF, et al. 2011. Diagnostic accuracy of point-of-care testing for acute coronary syndromes, heart failure and thromboembolic events in primary care: a cluster-randomised controlled trial. *BMC Family Practice* 12: 12

Vanderheyden M, Bartunek, Claeys G, et al. Head to head comparison of N-terminal pro-B-type natriuretic peptide and B-type natriuretic peptide in patients with/without left ventricular systolic dysfunction. *Clin Biochem*. 2006 Jun;39(6):640-5. Epub 2006 Mar 3.

Verdu JM, Comin-Colet J, Domingo M, Lupon J, Gomez M, Molina L, Casacuberta JM, Munoz MA, Mena A, Bruguera-Cortada J. 2012. Rapid point-of-care NT-proBNP optimal cut-off point for heart failure diagnosis in primary care. *Revista Espanola de Cardiologia* 65: 613-9

Villacorta H, Duarte A, Duarte NM, et al. The role of B-type natriuretic peptide in the diagnosis of congestive heart failure in patients presenting to an emergency department with dyspnea. *Arq Bras Cardiol.* 2002 Dec;79(6):569-72, 564-8.

Watson C, James S, O'Connell E, Gallagher J, O'Reilly J, Tallon E, Baugh J, O'Connell J, O'Shea D, Ledwidge M, McDonald K. 2016. Influence of diabetes on natriuretic peptide thresholds in screening for Stage B heart failure. *Biomarkers* 21: 538-43

Weekes AJ, Thacker G, Troha D, et al. Diagnostic Accuracy of Right Ventricular Dysfunction Markers in Normotensive Emergency Department Patients With Acute Pulmonary Embolism. Ann Emerg Med. 2016 Sep;68(3):277-91. doi: 10.1016/j.annemergmed.2016.01.027. Epub 2016 Mar 11.

Wei T, Zeng C, Chen L, et al. Bedside tests of B-type natriuretic peptide in the diagnosis of left ventricular diastolic dysfunction in hypertensive patients. Eur J Heart Fail. 2005 Jan;7(1):75-9.

Wieczorek SJ, Wu AH, Christenson R, et al. A rapid B-type natriuretic peptide assay accurately diagnoses left ventricular dysfunction and heart failure: a multicenter evaluation. Am Heart J. 2002 Nov;144(5):834-9.

Zapata L, Betbesé AJ, Roglan A, et al. Use of B-type natriuretic peptides to detect the existence and severity of diastolic dysfunction in non-cardiac critically ill patients: a pilot study. Minerva Anestesiol. 2014 Feb;80(2):194-203. Epub 2013 Oct 31.

Zhao SQ, Hu YM, Li Q, et al. The clinical value of rapid assay for plasma B-type natriuretic peptide in differentiating congestive heart failure from pulmonary causes of dyspnoea. Int J Clin Pract. 2008 Feb;62(2):214-20. Epub 2007 Dec 11.
